# Anesthetic action on the transmission delay between cortex and thalamus explains the beta-buzz observed under propofol anesthesia

**DOI:** 10.1371/journal.pone.0179286

**Published:** 2017-06-16

**Authors:** Meysam Hashemi, Axel Hutt, Darren Hight, Jamie Sleigh

**Affiliations:** 1 INRIA Grand Est - Nancy, Team NEUROSYS, Villers-lès-Nancy, France; 2 CNRS, Loria, UMR nō 7503, Vandoeuvre-lès-Nancy, France; 3 Université de Lorraine, Loria, UMR nō 7503, Vandoeuvre-lès-Nancy, France; 4 Aix Marseille Université, INSERM, INS, Institut de Neurosciences des Systèmes, Marseille, France; 5 German Meteorology Service, Offenbach am Main, Germany; 6 Department of Mathematics and Statistics, University of Reading, Reading, United Kingdom; 7 University of Auckland, Hamilton, New Zealand; University of Antwerp, BELGIUM

## Abstract

In recent years, more and more surgeries under general anesthesia have been performed with the assistance of electroencephalogram (EEG) monitors. An increase in anesthetic concentration leads to characteristic changes in the power spectra of the EEG. Although tracking the anesthetic-induced changes in EEG rhythms can be employed to estimate the depth of anesthesia, their precise underlying mechanisms are still unknown. A prominent feature in the EEG of some patients is the emergence of a strong power peak in the *β*–frequency band, which moves to the *α*–frequency band while increasing the anesthetic concentration. This feature is called the beta-buzz. In the present study, we use a thalamo-cortical neural population feedback model to reproduce observed characteristic features in frontal EEG power obtained experimentally during propofol general anesthesia, such as this beta-buzz. First, we find that the spectral power peak in the *α*– and *δ*–frequency ranges depend on the decay rate constant of excitatory and inhibitory synapses, but the anesthetic action on synapses does not explain the beta-buzz. Moreover, considering the action of propofol on the transmission delay between cortex and thalamus, the model reveals that the beta-buzz may result from a prolongation of the transmission delay by increasing propofol concentration. A corresponding relationship between transmission delay and anesthetic blood concentration is derived. Finally, an analytical stability study demonstrates that increasing propofol concentration moves the systems resting state towards its stability threshold.

## Introduction

General anesthesia is an indispensable tool in today’s medical surgery. It has been reported that in North America around 40 million patients receive general anesthesia for surgery [1, 2] each year. However, despite the widespread use of general anesthesia in today’s medical practice, its precise underlying mechanism is still unknown. During anesthesia, the anesthetics actions on the microscopic single neuron scale lead to specific changes in macroscopic-scale observables such as electroencephalogram (EEG) signals and the behavior of patients, such as loss of consciousness. For instance, the anesthetic *propofol* induces anesthesia accompanied by the increase in EEG spectral power in the *δ*– (0–4 Hz) and *α*– (8–15 Hz) frequency bands over the frontal head region [3, 4]. As the dose of propofol is slowly increased, high-amplitude, low-frequency oscillations emerge in EEG patterns [5, 6] and, in the phase of sedation, some subjects exhibit strong *β*-activity (15–30 Hz). Approaching the point of loss of consciousness (LOC), the slow EEG rhythms are more enhanced [7] and the EEG power peak in the *β*–band shifts to the *α*–frequency range [8–10]. This power shift from *β* to *α*–band is called *beta buzz*. Further increasing the anesthetic level causes burst suppression, which is characteristic of deep anesthesia [1, 11, 12].

In the present work, we employ a thalamo-cortical neural population feedback model to reproduce propofol-induced changes in EEG-spectral power within *δ*–, *α*- and *β*–frequency bands in the phase of sedation, i.e. before LOC occurs. To this end, to better understand the mechanisms underlying such specific features, we investigate the role of model parameters in the generation of spectral power peaks in these frequency bands. In this context, a previous work has considered the role of the synaptic [13] and extra-synaptic [4] connection strengths and addressed the question which thalamo-cortical sub-circuits contribute to the generation of *δ*– and *α*–activity [13]. In the present study, we focus on the anesthetic effect of synaptic time scales and the feedback delay between cortex and thalamus on spectral power modulations. We show how propofol modulates the synaptic time scales and the cortico-thalamo-cortical (CTC) transmission delay and how this modulation results in the induced *α*– and *δ*–peak and beta buzz pattern in EEG power spectrum. It is important to note that the current work does not aim to explain the underlying neurophysiological mechanism of characteristic EEG changes by rather complex dynamical models as some previous studies [8, 14]. In contrast, we propose a basic mechanism supported by a rather simple but neurophysiologically realistic thalamo-cortical population model.

Several previous modeling studies suggest that the time period of EEG *α*–rhythm depends on the transmission delay in the cortico-thalamic loop [15–18]. In these studies, similar to the study presented here, the transmission delay represents an effective population model entity that captures various delays along the pathway between thalamus and cortex. A recent study of Saggar *et al.* [19] has explored the effects of intensive meditation training on the parameters of a mean-field thalamo-cortical model. The authors report an observed reduction in the individual subject’s EEG *α*–frequency during meditation training. The corresponding model fit to the EEG power spectrum shows that the cortico-thalamic model feedback delay increases with training. This indicates an increase in transmission delay between cortical and thalamic neural structures [20].

Moreover, various model studies [21–23] have illustrated that the axonal transmission delays affect neural network interactions without changing the intrinsic input-output properties of individual neurons. Lumer *et al.* [21] concluded that the examination of the axonal transmission delays represents a simple way to test whether the observed oscillations result from a network property.

It is not well understood yet how anesthetic action on single neurons lead to characteristic modulations in EEG rhythms [24]. For instance, Ching *et al.* [8] have developed a thalamo-cortical model that suggests the importance of the thalamus in synchronous frontal *α*–activity in the EEG oscillations. In addition, it has been proposed that anesthetics disrupt neural synchrony including those which contribute to consciousness. Swindale [25] suggested that, in order to achieve synchronous firing in cortical neurons, the transmission time between any two cortical regions must be independent of path length over all the connected sites. Since the path length between different cortical regions varies between structures, the resulting delay variations between neural structures must be compensated for. Indeed, there is an enormous body of evidence for pathlength compensation in a variety of species and brain regions [26–28]. Moreover there is the hypothesis that anesthetic agents may affect the signal transmissions along the axonal fibers, in particular, the myelinated fibers [29, 30], which however is under discussion. Assuming that the physical properties of the axons such as their length and diameter remain unaffected by anesthetics, hypnotic agents must change the transmission delays along different axons [25]. Hudetz and Alkire have proposed that a mechanism for the preferential disruption of cortical feedback observed during general anesthesia may be that anesthetics impede axonal conduction along unmyelinated fibers [31].

In addition to the latter experimental and theoretical aspects, a closer look at the network structure along the pathways between cortex and thalamus reveals more evidence for an anesthetic effect on the transmission delay between both areas. Several sub-cortical structures are located along the pathways, such as the globus pallidus, the striatum and the hypothalamus [1]. Although there is evidence that these structures do not control the loss of consciousness directly [31], they may affect indirectly the way how sedation progresses. The neurons and synapses in the different neural structures along the diverse pathways are known to be affected by anesthetics, which may prolong the neurons and synapses time scales. Candidates for affected synaptic receptors are GABAergic, which are widely distributed in the brain and which are known to be sensitive to most anesthetic agents. It has been shown experimentally, that anesthetics prolong the response time of inhibitory GABAergic synapses by desensitisation in the range of several tens of milliseconds [32, 33].

To take into account the large number of possible anesthetic effects in a model, we assume that the delay between cortex and thalamus changes with anesthetic concentration and derive the mathematical relation between feedback delay and anesthetic blood concentration on the basis of the experimental data. We investigate how the EEG oscillatory frequency in *δ*– and *α*–frequency ranges could be varied by anesthetic effects on the transmission delays along pathways between cortex and thalamus during propofol-induced anesthesia.

## Materials and methods

### EEG acquisition during propofol-induced anesthesia

The EEG recording example is from a previously published study [34]. In brief, after obtaining the regional ethical committee approval, and written informed consent, ten healthy volunteers (aged 18 to 42 years) recruited from the department of Anaesthesia, Waikato District Health Board, Hamiton, New Zealand were studied. The EEG signal was recorded at a sampling rate of 256 Hz with 16 bit precision from a frontal electrode channel (FP7-FP1) using an Aspect A-1000 EEG machine (Aspect Medical Systems, Natick, MA, USA). Propofol was infused intravenously at 25 mg/min until the subject lost responsiveness to verbal command, which occurred typically after about 5 min. Low and high pass zero-phase butterworth filters were applied at 0.5 and 70 Hz respectively, as was a notch filter set at 50 Hz. Full methodological details are available in Ref. [34].

### Thalamo-cortical model

**Fig 1:**
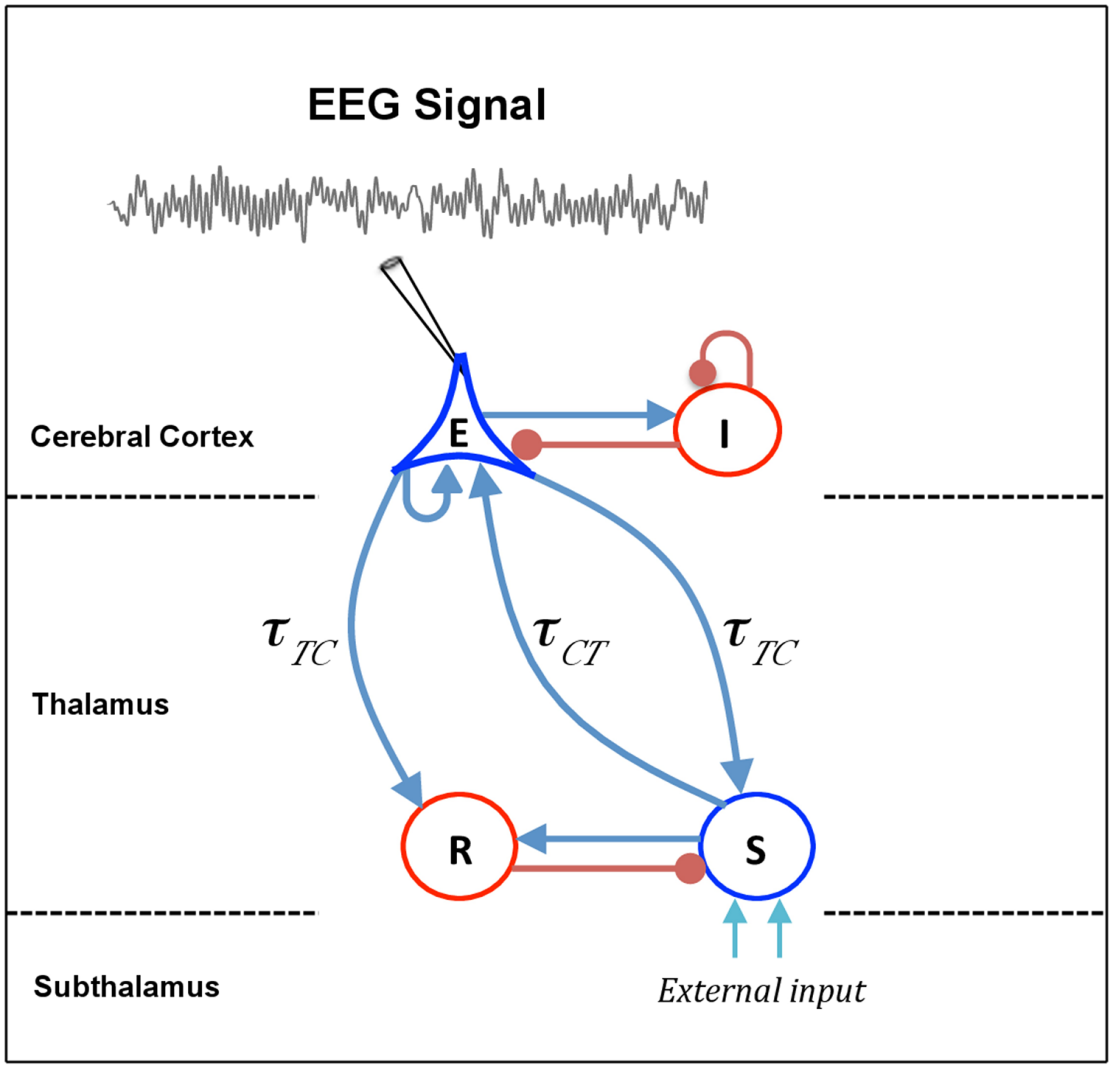
Schematic representation of a thalamo-cortical module. The model consists of four types of neural populations, namely, cortical excitatory (*E*) and inhibitory neurons (*I*), thalamo-cortical relay (*S*), and thalamic reticular neurons (*R*). The blue arrows show excitatory connections while the red connections with filled circle ends indicate inhibitory connections. The connections from cortex to thalamus is associated with the time delay *τ*_*TC*_, whereas the latency for signals in reverse direction is *τ*_*CT*_. The external input to the system originating from subthalamic areas is considered as a non-specific input to relay neurons.

In this section, we describe the thalamo-cortical neuronal population model used in this study, based on the work of Hutt and Longtin [35]. The anatomical structure of this model is similar to the models of Robinson *et al.* [18, 36, 37], however extends them by distinguishing the excitatory and inhibitory synapses as proposed by Hashemi *et al.* [13]. The model describes a network of four populations of neurons: cortical pyramidal neurons (E), cortical inhibitory neurons (I), thalamo-cortical relay neurons (S) and thalamic reticular nucleus (R), as shown schematically in Fig 1. The pyramidal neurons excite the cortical inhibitory neurons and receive inhibitory input from them. In addition, pyramidal neurons receive excitatory input from relay neurons and project back to the same nuclei. The connection from pyramidal neurons to relay cells (cortico-thalamic connection) is associated with time delay *τ*_*TC*_, whereas the latency for signals in reverse direction (thalamo-cortical connection) is *τ*_*CT*_. The reciprocal long-range excitatory interaction between pyramidal cells and relay neurons, called CTC loop in the following, would generates a positive feedback. However, the incessant excitation in this loop is prevented by the interposed inhibition to relay neurons, which originates from reticular nucleus. The reticular nucleus receives excitatory input from axon collaterals of pyramidal and relay neurons, while the cortex-reticular connection is associated with a constant time delay *τ*_*TC*_ [15, 38].

Note that several previous studies have considered a similar transmission delay in the thalamo-cortical and cortico-thalamic connections, and the time delay associated with CTC loop have been referred to as thalamo-cortical or cortico-thalamic delay. However, as presented in Ref. [39], axonal conduction from cortex to thalamus is much slower than in the reverse direction, i.e., *τ*_*TC*_ > *τ*_*CT*_. In this study, we distinguish the time delay in thalamo-cortical and cortico-thalamic connections. The conduction delay in the signal transmission from cortex to thalamus and back, denoted by *τ*, is referred to as CTC delay, i.e., *τ* = *τ*_*TC*_ + *τ*_*CT*_. Moreover, we consider an identical time delay for connections from cortex to relay and reticular cells. Thus, the time to traverse the loop from cortex to reticular nuclei, reticular to relay cells, and relay nuclei back to cortex (*esre* loop) is equivalent to CTC delay.

Consider spatially extended populations of excitatory and inhibitory neurons on the order of a few hundred micrometers. Furthermore, assume that the neural population of type *a* receives firing activity *P*_*e*_(*x*, *t*) and *P*_*i*_(*x*, *t*) at spatial location *x* and time *t*, which are originated from excitatory and inhibitory cells, respectively. Then, presynaptic population firing rates arrive at excitatory (*e*) or inhibitory (*i*) synapses and convert to the mean excitatory and inhibitory postsynaptic potentials $V_{a}^{e}\left(t\right)$ and $V_{a}^{i}\left(t\right)$, respectively, by the convolution [40]:

$$\begin{array}{c} V^{e,i}\left(x,t\right)=\int_{-\infty }^{t}h_{e,i}\left(t-t^{\prime }\right)P_{e,i}\left(x,t^{\prime }\right)dt^{\prime }, \end{array}$$ (1)

where the functions *h*_*e*_(*t*) and *h*_*i*_(*t*) indicate the mean synaptic response function of excitatory and inhibitory synapses, respectively, with

$$\begin{array}{c} h_{e}\left(t\right)=a_{e}\frac{\alpha_{e}\beta_{e}}{\beta_{e}-\alpha_{e}}\left(e^{-\alpha_{e}t}-e^{-\beta_{e}t}\right),\;\;h_{i}\left(t\right)=a_{i}f_{j}\left(p\right)\frac{\alpha_{i}\beta_{i}}{\beta_{i}-\alpha_{i}}\left(e^{-\alpha_{i}t}-e^{-\beta_{i}t}\right)\;. \end{array}$$ (2)

Here, 1/*α*_*e*_ and 1/*α*_*i*_ are the characteristic rise times of the response function for excitatory and inhibitory synapses, respectively, and 1/*β*_*e*_ and 1/*β*_*i*_ are the corresponding characteristic decay times. The parameters *a*_*e*_ and *a*_*i*_ stand for the average synaptic gains i.e., the level of excitation and inhibition, respectively. Furthermore, the function *f*_*j*_(*p*) reflects the action of propofol on inhibitory GABAergic receptors located in the cortex (j = C) and in the thalamus (j = T). The function *f*_*j*_ for *j* ∈ {*C*, *T*} will be described in the following subsection.

For convenience, the integral Eq (1) can be formulated to differential equations. Applying the second-order temporal derivative operators *L*_*e*,*i*_(∂/∂*t*) [15, 22, 37]

$$\begin{array}{c} {\hat{L}}_{k}\left(\partial /\partial t\right)=\frac{1}{\alpha_{k}\beta_{k}}\frac{\partial^{2}}{\partial t^{2}}+\left(\frac{1}{\alpha_{k}}+\frac{1}{\beta_{k}}\right)\frac{\partial }{\partial t}+1, \end{array}$$ (3)

with *k* = *e*, *i* on Eq (1) yields

$$\begin{array}{c} {\hat{L}}_{k}V^{k}\left(x,t\right)=a_{k}P_{k}\left(x,t\right). \end{array}$$ (4)

In each neural population, the mean population firing rate *P*_*e*,*i*_ depend on the effective mean potential and can be approximated by a sigmoidal function. Following our previous works [4, 13, 41], we identify the mean population firing rates with the transfer function *S*(*V*) derived from properties of type-I neurons [42]

$$\begin{array}{c} S\left(V_{a}\right)=Sig\left(V_{a},0\right)-Sig\left(V_{a},\rho \right), \end{array}$$ (5)

with

$$\begin{array}{c} Sig\left(V_{a},\rho \right)=\frac{S_{a}^{max}}{2}\left(1+erf\left(\frac{V_{a}-\theta_{a}-\rho \sigma_{a}^{2}}{\sqrt{2}\sigma_{a}}\right)\right)e^{-\rho \left(V_{a}-\theta_{a}\right)+\rho^{2}\sigma_{a}^{2}/2}, \end{array}$$ (6)

where $S_{a}^{max}$ denotes the maximum population firing rate, *θ*_*a*_ indicates the mean firing threshold of neurons, and *σ*_*a*_ is related to the standard deviation of firing thresholds in population of type *a*. In contrast to the standard sigmoid function *Sig*(*V*_*a*_, 0) assuming McCullogh-Pitts neurons [43], the model Eqs (5) and (6) assumes firing properties of type-I neuron and the transfer function Eq (5) is not anti-symmetric to its inflection point anymore [42, 43]. Note that *ρ* → ∞ yields the standard formulation assuming McCulloch-Pitts neurons with *Sig*(*V*, *ρ* → ∞) = 0. In the following, the mean firing rate function in the cortex *S*_*C*_[⋅] is considered to be different to the thalamic firing rate function *S*_*T*_[⋅] while, for the sake of simplicity, it is identical in both cortical structures and both thalamic structures.

### Modeling the propofol effect on neural populations

Now, there is a general agreement that anesthetics directly bind to specific molecular targets and there is not a single mechanism of action for all anesthetic agents [44–46]. Until today, various molecular targets have been identified to contribute to general anesthesia [24, 47]. Among different ligand-gated ion channels, GABA_A_ receptors are the most important molecular target for the action of many anesthetic drugs in the brain [48, 49]. According to the experimental evidence, the anesthetic propofol binds to both GABA_A_ and glutamatergic receptors. However, it has a much larger effect on GABA_A_ receptors as compared to its effect on AMPA and NMDA receptors [24, 47]. In addition, the experimental observations indicate that propofol increases the decay time constant of inhibitory GABA_A_ synapses, and hence increases the total charge transfer in these synapses [47]. Kitamura *et al.* [50] have shown that propofol has a negligible effect on the amplitude of synaptic response functions in cortical neurons, whereas experimental findings of Ying and Goldstein [51] in relay neurons in the thalamic ventrobasal complex indicate a markedly increase in amplitude, decay time, and thus charge transfer of GABA_A_ receptors during propofol application. It is important to point out that GABA_A_ receptors are also found outside the synapse. Tonic inhibitory currents at these extra-synaptic GABA_A_ receptors are also potentiated by general anesthetics [52, 53]. Although extra-synaptic inhibition affects the EEG during anesthesia [4], the current work neglects this effect for simplicity.

In order to integrate the mentioned experimental observations into the neural model, the effect of propofol on GABAergic receptors is modeled by $\beta_{i}\to \beta_{i}^{0}/p$ with *p* ≥ 1 [54], where $\beta_{i}^{0}$ denotes the decay rate of inhibitory synapses in the absence of propofol (*p* = 1). Here, the factor *p* indicates the on-site concentration of propofol in the neural populations, and *p* = 1 reflects zero anesthetic concentration i.e., the baseline condition. The administration of propofol (*p* > 1) leads to a decrease in the decay rate constant *β*_*i*_ of inhibitory synapses and hence an increase in the charge transfer of these synapses. Similar to our previous work [13], in order to distinguish the propofol effect on the amplitude of inhibitory synaptic response function in the cortex and in the thalamus, the functions *f*_*C*_(*p*), and *f*_*T*_(*p*) are defined as

$$\begin{array}{ccc} f_{C}\left(p\right) & = & a_{i}\Gamma \left(\alpha ,\beta_{i}^{0}\right)/\Gamma \left(\alpha ,\beta_{i}\right), \end{array}$$ (7)

$$\begin{array}{ccc} f_{T}\left(p\right) & = & a_{i}A_{r}\left(p\right)\Gamma \left(\alpha ,\beta_{i}^{0}\right)/\Gamma \left(\alpha ,\beta_{i}\right), \end{array}$$ (8)

with

$$\begin{array}{c} \Gamma \left(\alpha ,\beta \right)=\frac{\alpha \beta }{\alpha -\beta }\left[{\left(\alpha /\beta \right)}^{\frac{-\beta }{\alpha -\beta }}-{\left(\alpha /\beta \right)}^{\frac{-\alpha }{\alpha -\beta }}\right]\;. \end{array}$$

In accordance with the above mentioned experimental observations, *f*_*C*_(*p*) fixes the maximum of the cortical response function, whereas the function *A*_*r*_(*p*) allows the inhibitory response amplitude to change for thalamic receptors. An optimal fit to experimental data has revealed *A*_*r*_(*p*)≈*p*^0.42^ [13].

Taken together, the propofol effects on the thalamo-cortical module shown in Fig 1 are modeled as a decrease in decay rate of inhibitory synaptic response function by *β*_*i*_ → *β*_*i*_/*p*, an increase in the charge transfer in cortical inhibitory transmissions *I* → *E* and *I* → *I* by the function *f*_*C*_(*p*), and an increase in the charge transfer in thalamic inhibitory transmission *R* → *S* by the function *f*_*T*_(*p*). Note that the propofol prolongs the decay phases only in inhibitory synapses, while other synaptic rates are unaffected by propofol.

### Model equations

The mean postsynaptic potentials $V_{a}^{c}$, evoked at excitatory (*c* = *e*) and inhibitory (*c* = *i*) synapses, for *a* ∈ {*E*, *I*, *R*, *S*} in the cortical pyramidal neurons (*E*), cortical inhibitory neurons (*I*), the thalamo-cortical relay neurons (*S*) and thalamic reticular neurons (*R*) obey

$$\begin{array}{ccc} {\hat{L}}_{e}V_{E}^{e}\left(x,t\right) & = & K_{EE}\left(x\right)*S_{C}\left[V_{E}^{e}\left(x,t\right)-V_{E}^{i}\left(x,t\right)\right]+\\ & & K_{ES}\left(x\right)*S_{T}\left[V_{S}^{e}\left(x,t-\tau_{CT}\right)-V_{S}^{i}\left(x,t-\tau_{CT}\right)\right],\\ {\hat{L}}_{i}V_{E}^{i}\left(x,t\right) & = & f_{C}\left(p\right)K_{EI}\left(x\right)*S_{C}\left[V_{I}^{e}\left(x,t\right)-V_{I}^{i}\left(x,t\right)\right],\\ {\hat{L}}_{e}V_{I}^{e}\left(x,t\right) & = & K_{IE}\left(x\right)*S_{C}\left[V_{E}^{e}\left(x,t\right)-V_{E}^{i}\left(x,t\right)\right],\\ {\hat{L}}_{i}V_{I}^{i}\left(x,t\right) & = & f_{C}\left(p\right)K_{II}\left(x\right)*S_{C}\left[V_{I}^{e}\left(x,t\right)-V_{I}^{i}\left(x,t\right)\right],\\ {\hat{L}}_{e}V_{S}^{e}\left(x,t\right) & = & K_{SE}\left(x\right)*S_{C}\left[V_{E}^{e}\left(x,t-\tau_{TC}\right)-V_{E}^{i}\left(x,t-\tau_{TC}\right)\right]+I\left(x,t\right),\\ {\hat{L}}_{i}V_{S}^{i}\left(x,t\right) & = & f_{T}\left(p\right)K_{SR}\left(x\right)*S_{T}\left[V_{R}^{e}\left(x,t\right)\right],\\ {\hat{L}}_{e}V_{R}^{e}\left(x,t\right) & = & K_{RE}\left(x\right)*S_{C}\left[V_{E}^{e}\left(x,t-\tau_{TC}\right)-V_{E}^{i}\left(x,t-\tau_{TC}\right)\right]+\\ & & K_{RS}\left(x\right)*S_{T}\left[V_{S}^{e}\left(x,t\right)-V_{S}^{i}\left(x,t\right)\right], \end{array}$$ (9)

where *K*_*ab*_(*x*) ∗ *S*[*V*(*x*, *t*)] = ∫_Ω_*K*_*ab*_(*x* − *y*)*S*[*V*(*y*, *t*)]*dy*. The spatial kernel functions *K*_*ab*_(*x* − *y*) reflect the synaptic connection strengths in population *a* originating from population *b* in the spatial domain Ω. According to previous studies, we assume that the EEG can be described by a spatially constant neural population activity [15, 55] in a good approximation. In addition, *K*_*ab*_(*x* − *y*) = *K*_*ab*_*δ*(*x* − *y*) with the Dirac function *δ*(⋅) and thereby *K*_*ab*_(*x*) ∗ *S*[*V*(*x*, *t*)] = *K*_*ab*_*S*[*V*(*x*, *t*)]. The additional activity *I*(*x*, *t*) introduces an external input to the system, which may originate from other neural populations. Here the external input is considered as a non-specific input to relay neurons

$$\begin{array}{c} I\left(x,t\right)=I_{0}+\xi \left(x,t\right), \end{array}$$ (10)

where *I*_0_ = *const* is the input mean value, and *ξ*(*x*, *t*) indicates zero-mean Gaussian white noise with

$$\begin{array}{c} \left\langle \xi \left(x,t\right)\right\rangle =0,\;\;\left\langle \xi \left(x,t\right)\xi \left(x^{\prime },t^{\prime }\right)\right\rangle =2\kappa \delta \left(t-t^{\prime }\right)\delta \left(x-x^{\prime }\right), \end{array}$$ (11)

and *κ* is the intensity of the noise and 〈〉 denotes the ensemble average.

The nominal values the model parameter are displayed in Table 1 in the absence of anesthetics.

**Table 1:** Model parameters, their symbols, and nominal values for two parameter sets.

Parameter	Symbol	Nominal value
Maximum firing-rate of cortical population	$S_{C}^{max}$	130 Hz
Maximum firing-rate of thalamic population	$S_{T}^{max}$	100 Hz
Mean firing threshold of cortical population	$V_{C}^{th}$	25 mV
Mean firing threshold of thalamic population	$V_{T}^{th}$	25 mV
Firing rate variance	*σ*	10 mV
Type-I population effect constant	*ρ*	0.05 mV^−1^
Excitatory synaptic rise rate	*α*_*e*_	1000 *s*^−1^
Excitatory synaptic decay rate	*β*_*e*_	100 *s*^−1^
Inhibitory synaptic rise rate	*α*_*i*_	500 *s*^−1^
Inhibitory synaptic decay rate	*β*_*i*_	10 *s*^−1^
Excitatory synaptic gain	*a*_*e*_	1 mVs
Inhibitory synaptic gain	*a*_*i*_	1 mVs
Synaptic strength from E to E neurons	*K*_*EE*_	0.1 mVs
Synaptic strength from E to I neurons	*K*_*IE*_	0.3 mVs
Synaptic strength from E to S neurons	*K*_*SE*_	0.8 mVs
Synaptic strength from E to R neurons	*K*_*RE*_	0.2 mVs
Synaptic strength from I to I neurons	*K*_*II*_	0.2 mVs
Synaptic strength from I to E neurons	*K*_*EI*_	0.6 mVs
Synaptic strength from S to E neurons	*K*_*ES*_	0.8 mVs
Synaptic strength from S to R neurons	*K*_*RS*_	0.1 mVs
Synaptic strength from R to S neurons	*K*_*SR*_	0.8 mVs
Constant external input	*I*_0_	0.1 mV
Intensity of external thalamic noise	*κ*	0.5 mV
Cortico-thalamic delay transmission	*τ*_*TC*_	60 ms
Thalamo-cortical delay transmission	*τ*_*CT*_	20 ms
Time delay transmission in CTC loop	*τ*	80 ms

### Theoretical power spectrum

The set of model Eq (9) can be written as

$$\begin{array}{c} \hat{L}\left(\partial /\partial t\right)X\left(t\right)=\Phi \left(X\left(t\right)\right)+\Psi \left(X\left(t-\tau_{TC}\right)\right)+\Xi \left(X\left(t-\tau_{CT}\right)\right)+I\left(t\right), \end{array}$$ (12)

where those terms that have delays are separated from those terms without delays.

Here, $\hat{L}\left(\partial /\partial t\right)\in R^{N\times N}$ is the diagonal matrix operator including the temporal operators ${\hat{L}}_{e,i}$ (c.f. Eq (3)), and $X\left(t\right)\in R^{N}$ indicates the activity variable vector with dimension *N*. Considering Eq (9), $X\left(t\right)={\left(V_{E}^{e},V_{E}^{i},V_{I}^{e},V_{I}^{i},V_{S}^{e},V_{S}^{i},V_{R}^{e}\right)}^{⊤}\in R^{N}$, where *N* = 7. In addition, the external input is given by ***I***(*t*) = ***I***_0_ + **ξ**(*t*), where ***I***_0_ = (0, 0, 0, 0, *I*_0_, 0, 0)^⊤^ and **ξ**(*t*) = (0, 0, 0, 0, *ξ*(*t*), 0, 0)^⊤^. Here, the high index ⊤ denotes the transposed vector or matrix.

Now, the time-independent resting state $X_{0}\in R^{N}$ with $\hat{L}\left(\partial /\partial t\right)=\hat{L}\left(0\right)$ can be obtained from ***X***_0_ = **Φ**(***X***_0_) + **Ψ**(***X***_0_) + **Ξ**(***X***_0_) + ***I***_0_. Linearizing Eq (12) about the resting state ***X***_0_ yields

$$\begin{array}{c} \hat{L}\left(\partial /\partial t\right)Y\left(t\right)=AY\left(t\right)+BY\left(t-\tau_{TC}\right)+CY\left(t-\tau_{CT}\right)+\xi \left(t\right), \end{array}$$ (13)

where ***Y***(*t*) = ***X***(*t*) − ***X***_0_ denotes the small deviations from the resting state of the system, and ***A***, ***B***, $C\in R^{N\times N}$ are constant quadratic matrices such that ***A*** ≡ **J**_**Φ**_(***X***_**0**_), ***B*** ≡ **J**_**Ψ**_(***X***_**0**_) and ***C*** ≡ **J**_**Ξ**_(***X***_**0**_), where **J**_**Φ**_(***X***_0_), **J**_**Ψ**_(***X***_0_) and **J**_**Ξ**_(***X***_0_) are the Jacobian matrices of functions **Φ**, **Ψ** and **Ξ**, respectively, computed at the resting state ***X***_0_.

By considering the specific choice of external input to the *j*-th element of the activity variable, it can be shown that the power spectrum of *i*-th element just depends on one matrix component of the matrix Green’s function (see S1 Appendix)

$$\begin{array}{c} P_{i}\left(\omega \right)=2\kappa \sqrt{2\pi }{\left|{\tilde{G}}_{i,j}\left(\omega \right)\right|}^{2}, \end{array}$$ (14)

where *ω* denotes the complex angular frequency.

In the following, to compute the EEG-spectral power, we assume that the EEG is generated by the population activity of pyramidal cortical cells $V_{E}^{e}$ [18, 56, 57]. Moreover, it has been extensively demonstrated that linear approximations about a time-invariant resting state are sufficient for the prediction of EEG variables [15, 37, 58–60]. By applying the aforementioned approach, one can compute analytically the EEG power spectrum under the assumption of spatial homogeneity [35] and gains [13]

$$\begin{array}{c} P_{E}\left(\omega \right)=2\kappa \sqrt{2\pi }{\left|{\tilde{G}}_{1,5}\left(\omega \right)\right|}^{2}\;. \end{array}$$ (15)

Here, the external input drives directly the excitatory postsynaptic potential $V_{S}^{e}$ in relay neurons (see S2 Appendix for definition of $\tilde{G}\left(\omega \right)$ components). It is important to point out that, although two time delays *τ*_*TC*_ and *τ*_*CT*_ appear in the system equations the EEG spectral power depends on the their sum, i.e., the CTC transmission delay (cf. section Constrains on system stability).

### Stability analysis of the linear model

In the following, to investigate the stability of the system resting state, we neglect the external noisy input *ξ*(*t*), since the asymptotic stability of linear systems does not depend on small external perturbations. Theory of linear delayed systems states that the solution of Eq (13) is a linear superposition of single mode solutions ***Y***(*t*) = ***C****e*^*λt*^, λ∈C and the characteristic equation for the eigenvalue *λ* can be written as

$$\begin{array}{c} P\left(\lambda \right)+e^{-\lambda \tau }Q\left(\lambda \right)=0\;. \end{array}$$ (16)

The terms *P*(*λ*) and *Q*(*λ*) are polynomial in *λ*. Eq (16) is transcendental and has an infinite number of complex roots *λ*.

The solution of Eq (13) is asymptotically stable if and only if all the characteristic roots *λ* have negative real parts, i.e., *Re*(*λ* < 0). The solution is called unstable if there exists a root with positive real part. Although a large number of studies have been devoted to obtain exact stability conditions for delayed differential equations such as Eq (13), this problem is still unsolved for a wide class of model types [61–63]. To this end, typically numerical methods are employed such as numerical integration methods [64, 65], employing the Lambert function [66, 67], the discretization of the solution operator as implemented in the software package DDE-BIFTOOL [68, 69], or discretising the infinitesimal generator as implemented in the software package TRACE-DDE [70, 71]. In order to obtain numerically the characteristic roots of a transcendental equation, we have used the spectral discretization approach based on the discretization of the PDE-representation [65, 72]. This method turns the infinite-dimensional problem of the numerical computation of the characteristic roots into a finite-dimensional eigenvalue problem for a suitable matrix [70, 71], see S3 Appendix.

We also employ an analytical approach to obtain stability conditions for the thalamo-cortical system, and we compare them to the numerical solutions. The method used in this study is analytically tractable, in particular for high-order equations. This method treats the duration of the time delay as a bifurcation parameter to seek whether or not varying the delay can change the stability characteristics of the resting state. Increasing the delay value, a bifurcation occurs if the resting state becomes unstable and the characteristic roots *λ* traverse the imaginary axis. In this approach, finding a delay value which induces instability in the system reduces to the problem of finding positive real roots of the related polynomial equation. The method is based on the following statement (for the details see Ref. [73]):

Consider a system of delay differential equations whose associated characteristic equation is given by
  $$\begin{array}{c} \sum_{n=0}^{N}a_{n}\lambda^{n}+e^{-\lambda \tau }\sum_{n=0}^{M}b_{n}\lambda^{n}=0. \end{array}$$
  Assume that the resting state is stable in the absence of delay (*τ* = 0). Then, increasing delay value there exists a critical delay τ* > 0 for which the resting state becomes unstable if and only if the characteristic polynomial P(Ω) defined as
  $$\begin{array}{c} {\left(\sum_{n}{\left(-1\right)}^{n}a_{2n}\Omega^{n}\right)}^{2}+\Omega {\left(\sum_{n}{\left(-1\right)}^{n}a_{2n+1}\Omega^{n}\right)}^{2}={\left(\sum_{n}{\left(-1\right)}^{n}b_{2n}\Omega^{n}\right)}^{2}+\Omega {\left(\sum_{n}{\left(-1\right)}^{n}b_{2n+1}\Omega^{n}\right)}^{2}, \end{array}$$
  has a positive real root Ω* = (ω*)^2^, where *λ* = *iω*, ω∈R.

Therefore, at first one has to determine the stability of the resting state in the absence of delay (*τ* = 0). If the resting state is stable when *τ* = 0, then for *τ* > 0, we investigate whether P(Ω) has any positive real root. If P(Ω) has not any positive real root, increasing *τ* does not lead to any change in the stability of the resting state. Conversely, if P(Ω) has a positive real root, there is a critical value for delay *τ* for which causes a bifurcation, and destabilizes the resting state of the system.

To determine whether a characteristic polynomial has any positive real root or not, it is not necessary to compute the roots explicitly. Instead, it is merely sufficient to apply simpler approaches such as Descartes’ Rule of Signs. This method determines the maximum number of positive and negative real roots of a polynomial equation. If the number of sign changes is odd, a positive real root is guaranteed. If, however, the number of sign changes is even, one has to use a more general approach such as the so-called Sturm sequences (see Ref. [73]).

### Identification of the EEG spectral peaks

To compute the peaks of EEG power spectrum, we relate the maxima of spectral power to the roots of its characteristic equation i.e., the roots of denominator of system Green’s function. Considering *λ* = *γ* ± 2*πiν* as a characteristic root, *γ* and *ν* illustrate the damping rate and the frequency of corresponding power peak, respectively. The *δ*– and *α*–power peaks are defined as *ν* ∈ (0–4 Hz) and *ν* ∈ (8–15 Hz), respectively.

Note that in the case of large damping rate in a specific frequency band, we may not observe the related spectral power peak in the plotted power spectrum, although the imaginary parts of characteristic roots are not necessarily equal to zero. In this case, the first derivative of the power spectrum has no roots in that frequency range. In Results section, we determine the spectral power peaks by computing the system characteristic roots and for the cases of large damping rates, we also compute the roots of first derivative of power spectrum.

## Results

### The effect of thalamo-cortical time-scales on EEG power peaks

At first we investigate how the spectral power peaks in *δ*– and *α*–frequency ranges depend on the thalamo-cortical time scales, i.e. synaptic time constants and the CTC transmission delay. Then, by taking into account the impact of time scale parameters of the system on the model behavior, we show that the model is able to reproduce well the observed specific features in frontal EEG power spectrum during propofol-induced anesthesia.

**Fig 2:**
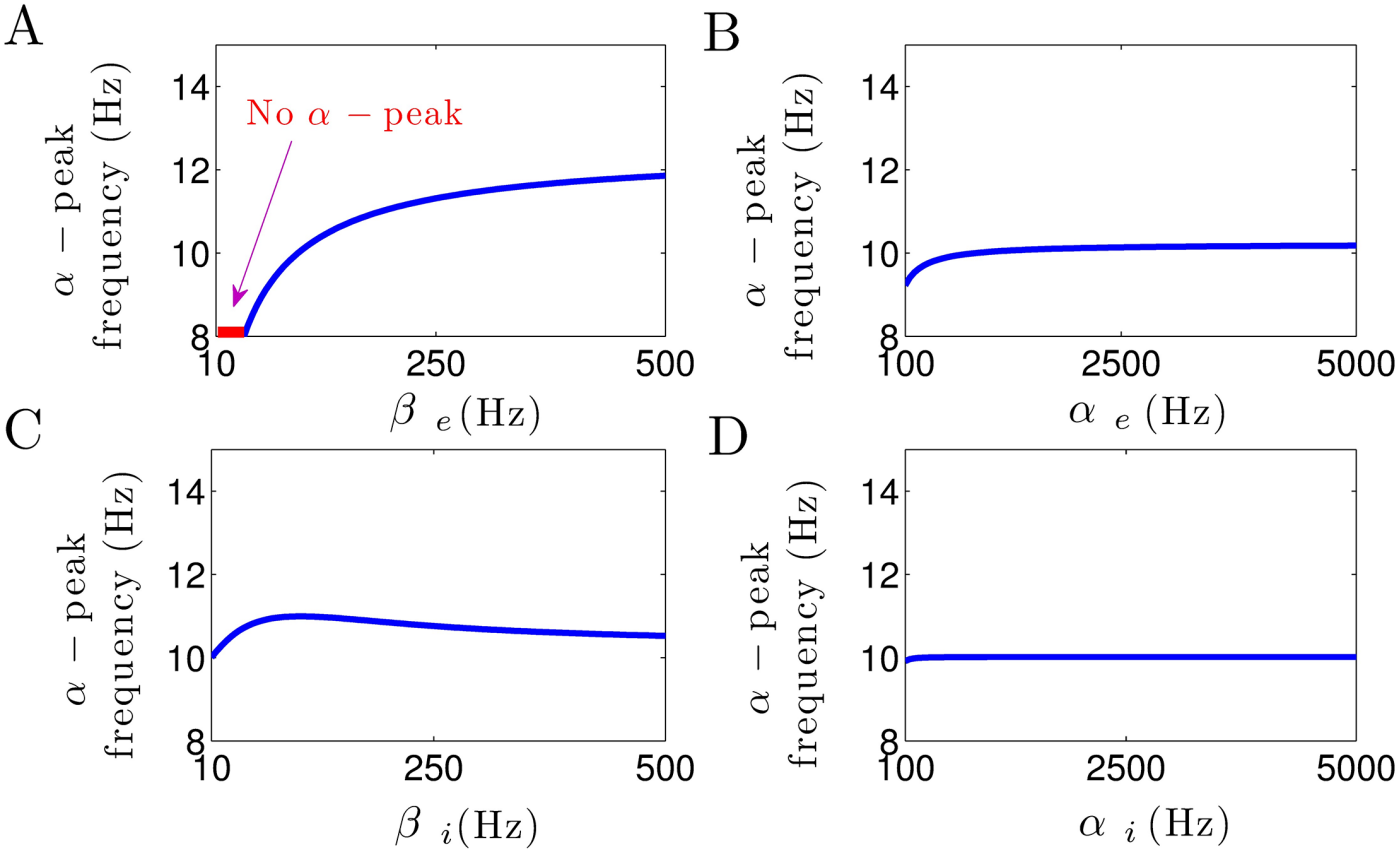
The *α*–peak depend on synaptic time constants. (A) Increasing *β*_*e*_ increases the *α*–power peak frequency. The red color shows the region *β*_*e*_ < 40 Hz where no spectral power peak in the *α*–range can be observed. (B) The *α*–peak frequency remains more or less unchanged by the increase in *α*_*e*_. (C) The increase in *β*_*i*_ has an insignificant effect on *α*–power peak frequency, whereas (D) it remains unaffected by the increase in *α*_*i*_. In all panels, unchanged parameters are taken from Table 1.

Fig 2 shows the impact of increasing excitatory and inhibitory synaptic rate constants on the approximate frequency of the *α*–power peak. Fig 2A illustrates that the decay rate constant of excitatory synapses *β*_*e*_ heavily affects the location of *α*–power peak frequency. Note that for small excitatory decay rates *β*_*e*_ < 40 Hz no *α*–band spectral power peak is present. This result reveals the crucial role of the excitatory synaptic decay time in the generation of *α*–power peak frequency. Fig 2B–2D illustrate well that the rise rate of excitatory synapses *α*_*e*_, and both rise and decay rate of inhibitory synapses, *α*_*i*_ and *β*_*i*_, respectively, have an insignificant effect on the *α*–power peak frequency.

**Fig 3:**
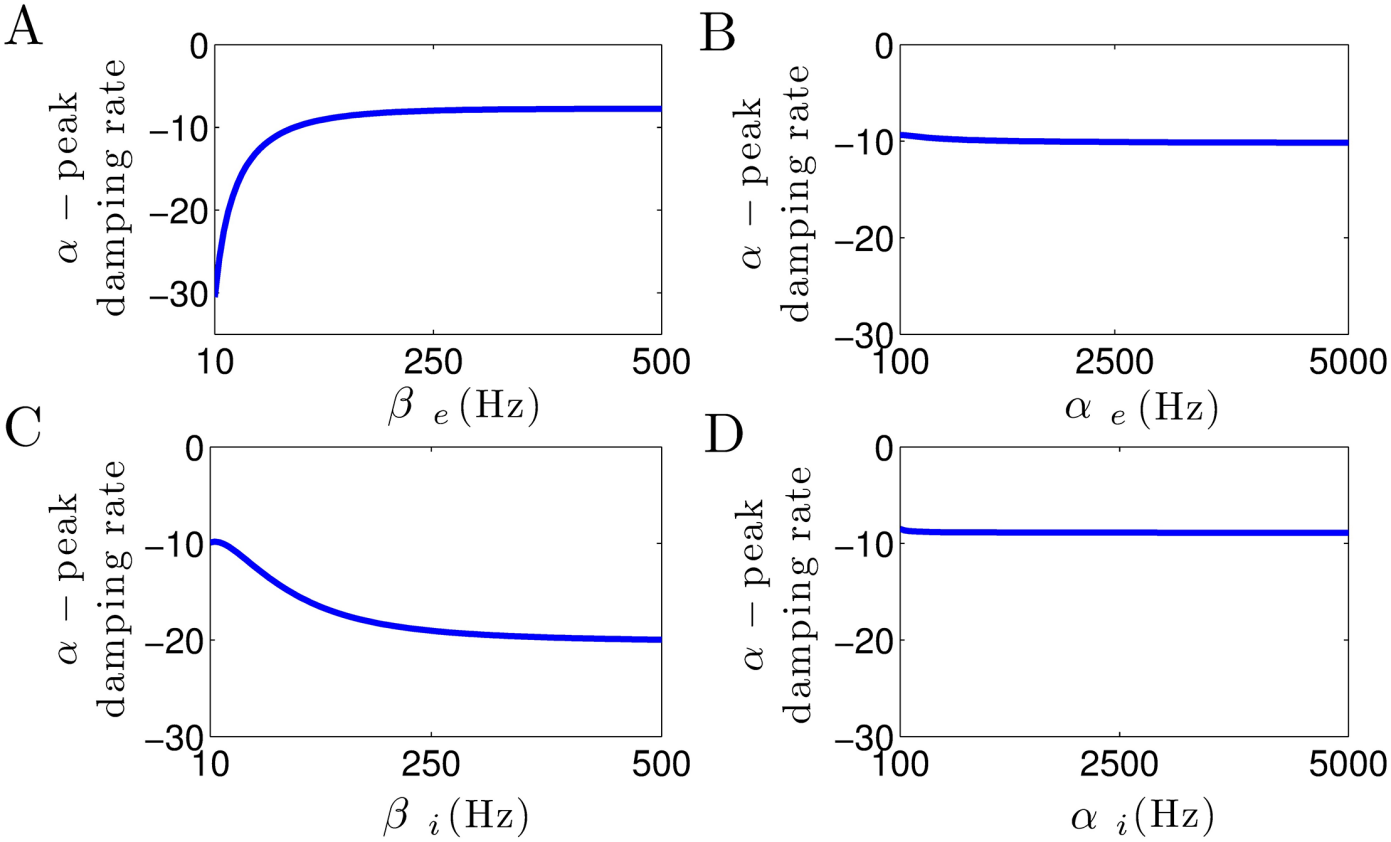
Modulation of damping rate of *α*–activity subjected to excitatory and inhibitory synaptic time constants. (A) The *α*–peak damping rate considerably increases by the increase in *β*_*e*_, whereas (C) it slightly decreases as *β*_*i*_ increases. (B, D) Damping rate of *α*–activity remains unaffected by the increase in *α*_*e*_ and *α*_*i*_. In all panels, unchanged parameters are taken from Table 1.

Fig 3 shows how the damping rate of *α*–activity is affected by the increase in excitatory and inhibitory synaptic time constants. In Fig 3A, it can be seen that the damping rate of *α*–activity considerably increases with increasing the excitatory decay rate constant *β*_*e*_. This leads to an increase in *α*–power, since the system moves toward an instability point. Note that for small values of *β*_*e*_, due to the high damp of *α*–oscillation, no spectral power peak can be observed in the corresponding frequency band (cf. Fig 2A). Moreover, in Fig 3C, we observe that the *α*–peak damping rate slightly decreases as the the inhibitory decay rate *β*_*i*_ increases, whereas Fig 3B and 3D illustrate that the damping rate of *α*–activity remains unaffected by the increase in excitatory and inhibitory rise rates *α*_*e*_ and *α*_*i*_.

Briefly, these results demonstrate that the excitatory decay rate constant plays a critical role in *α*–power generation, whereas the rise time constants of excitatory and inhibitory synapses do not affect the *α*–power activity.

**Fig 4:**
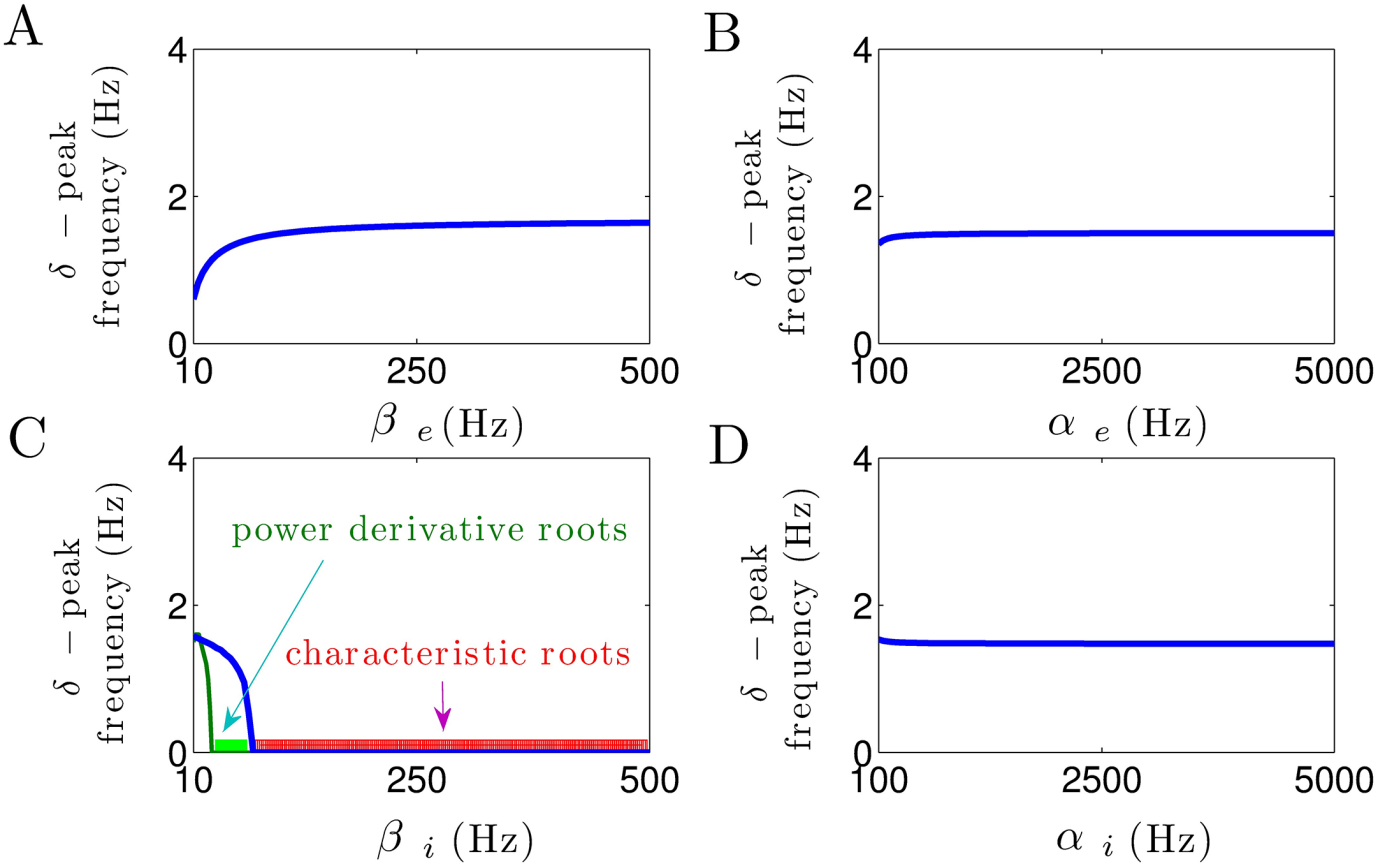
Modulation of *δ*–peak frequency subjected to excitatory and inhibitory synaptic time constants. (A) The *δ*–peak frequency slightly increases with the increase in *β*_*e*_. (B, D) Excitatory and inhibitory rise time constants do not affect the *δ*–peak frequency. (C) Inhibitory decay time constant heavily affects the frequency of *δ*–activity. Note that for 30 < *β*_*i*_ < 75 Hz, the imaginary parts of characteristic roots in *δ*–range are not equal to zero (blue line), but the derivative of power spectrum has no roots in *δ*–frequency range (illustrated by green color). For *β*_*i*_ > 75 Hz, the imaginary parts of characteristic roots in *δ*–range are zero and also the spectral power derivative has no roots (illustrated by red color). In all panels, unchanged parameters are taken from Table 1.

In a similar manner, Fig 4 illustrates how the peak frequency of *δ*–activity depends on the excitatory and inhibitory synaptic time constants. Increasing the excitatory decay rate slightly increases the *δ*–peak frequency, cf. Fig 4A. Moreover, Fig 4C shows that a small increase in the inhibitory decay rate constant *β*_*i*_ at small values yields a rapid decrease in *δ*–power peak frequency. Most importantly, *β*_*i*_ > 75 Hz makes the power peak frequency at *δ*–range vanish (i.e., *δ*–peak frequency is zero since the imaginary parts of characteristic roots in *δ*–band are zero, as illustrated by red color). Note that for 30 < *β*_*i*_ < 75 Hz, the imaginary parts of characteristic roots in *δ*–range are not equal to zero (blue line). However, due to the large values of corresponding damping rates, no spectral power peak in *δ*–band can be observed (i.e., the the first derivative of the power spectrum has no roots in *δ*–frequency range, as illustrated by green color).

Furthermore, in Fig 4B and 4D, we observe that the frequency of spectral power peak within the *δ*–band remains constant with respect to parameters *α*_*e*_ and *α*_*i*_.

**Fig 5:**
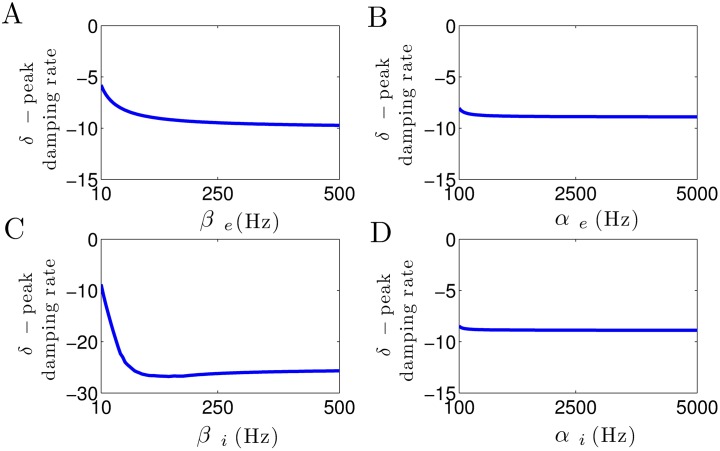
Modulation of damping rate of *δ*–activity subjected to excitatory and inhibitory synaptic time constants. (A) The *δ*–peak damping rate slightly decreases by the increase in *β*_*e*_, whereas (C) it considerably decreases as *β*_*i*_ increases. (B, D) Damping rate of *δ*–activity remains constant with respect to parameters *α*_*e*_ and *α*_*i*_. In all panels, unchanged parameters are taken from Table 1.

Fig 5 shows the effect of increase in synaptic time constants on the damping rate of *δ*–activity. In Fig 5A, we observe that the *δ*–peak damping rate slightly decreases with the increase in excitatory decay rate *β*_*e*_, whereas it considerably decreases as the inhibitory decay rate *β*_*i*_ increases (see Fig 5C). Note that due to the high level of *δ*–power damp for *β*_*i*_ > 30 Hz, no spectral power peak is present in *δ*–frequency band (i.e., the derivative of power spectrum has no roots in *δ*–band, as shown in Fig 4C). Furthermore, from Fig 5B and 5D, it can be seen that the excitatory and inhibitory rise time constants *α*_*e*_ and *α*_*i*_, respectively, do not affect the damping rate of *δ*–power activity.

In sum, these results reveal the critical role of inhibitory decay rate constant in *δ*–power generation, whereas excitatory and inhibitory rise time constants have no effect on *δ*–power modulation.

**Fig 6:**
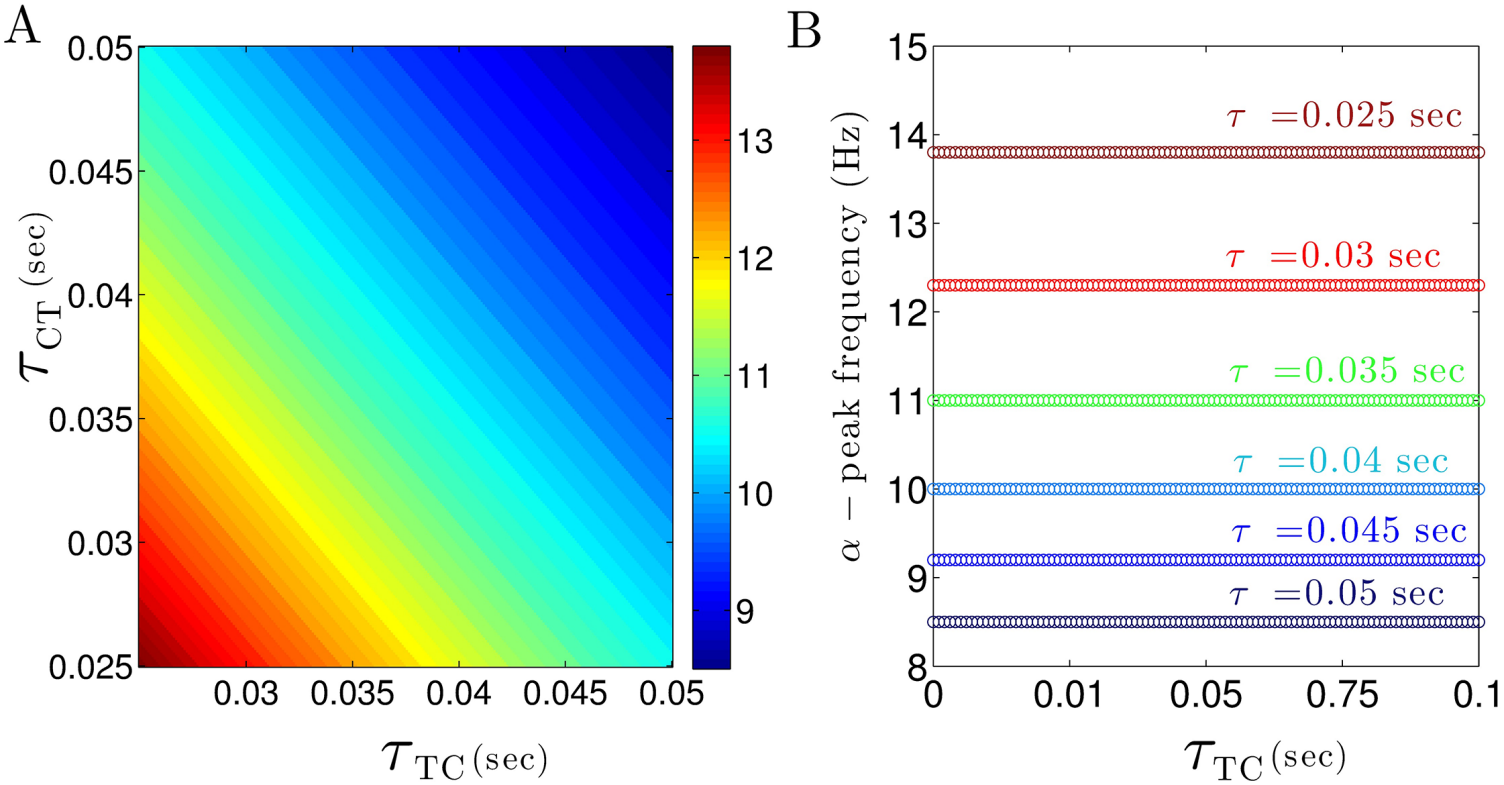
*α*–peak frequency depends on feedback delays in the thalamo-cortical system. (A) The frequency of *α*–power subjected to thalamo-cortical (*τ*_*CT*_) and cortico-thalamic (*τ*_*TC*_) delays. (B) The frequency of *α*–power for different values of the CTC delay *τ* and *τ*_*TC*_ with *τ*_*CT*_ = *τ* − *τ*_*CT*_. In all panels, unchanged parameters are taken from Table 1.

Besides the synaptic time scales, the delay times of cortico-thalamic and thalamo-cortical connections *τ*_*CT*_ and *τ*_*TC*_, respectively, may affect the spectral power peaks. Fig 6 presents the *α*–power peak frequency with respect to the thalamo-cortical (*τ*_*CT*_) and cortico-thalamic (*τ*_*TC*_) transmission delays. In Fig 6A, we observe that the frequency decreases with increasing delay times and the *α*–peak frequency is constant on a line *τ*_*CT*_ + *τ*_*TC*_ = const. This relation is validated in Fig 6B revealing that the *α*–peak frequency depends on the total CTC delay *τ* = *τ*_*CT*_ + *τ*_*TC*_ only. Our simulations illustrate the similar results for *δ*–peak frequency (not shown). This finding is validated in section Constrains on system stability.

**Fig 7:**
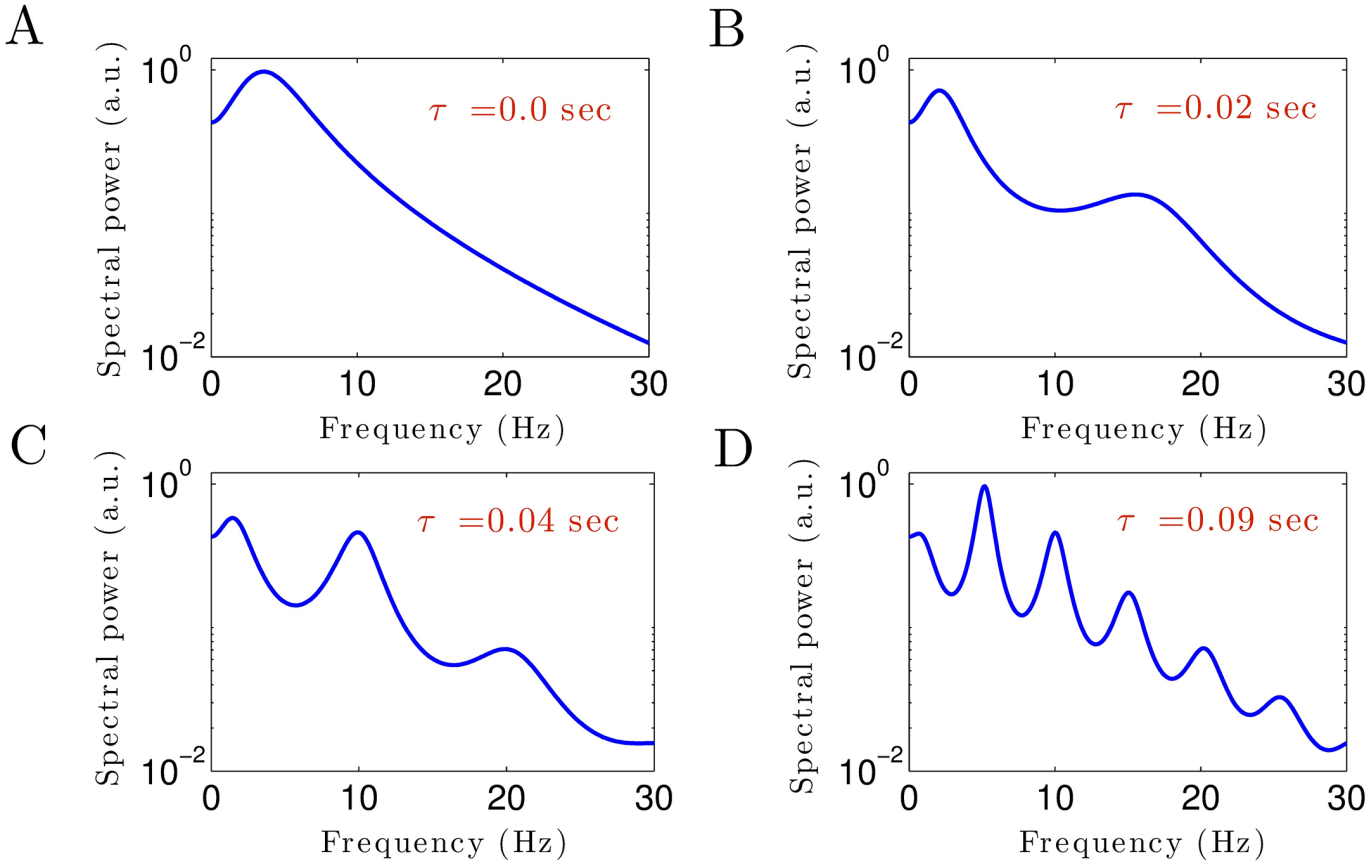
Theoretical EEG power spectra (Eq (15)) for different values of the CTC time delay. In all panels, unchanged parameters are taken from Table 1.

To illustrate further how CTC delay times affect the power spectrum, Fig 7 shows theoretical EEG power spectra for different values of CTC delay *τ*. For no CTC delay, the power spectrum exhibits a peak in the *δ*–frequency range, but no prominent *α*–activity (Fig 7A). Increasing the delay time generates activity at high frequencies in the *α*–band (at about 15 Hz for *τ* = 0.02 sec in Fig 7B) whose power peak increases in magnitude and decreases in frequency with even larger delay times (Fig 7C). In addition, the second harmonic of the *α*–peak occur in the *β*–frequency range. Essentially even large values of time delay (Fig 7D) generates multiple power peaks in all frequency bands. In contrast, the CTC delay affects much less the power peak of *δ*–activity. These results show that the existence and frequencies of the spectral power peak of *α*–activity critically depends on the transmission delay in the CTC circuit, whereas the presence of *δ*–power peak is independent of CTC delay values.

**Fig 8:**
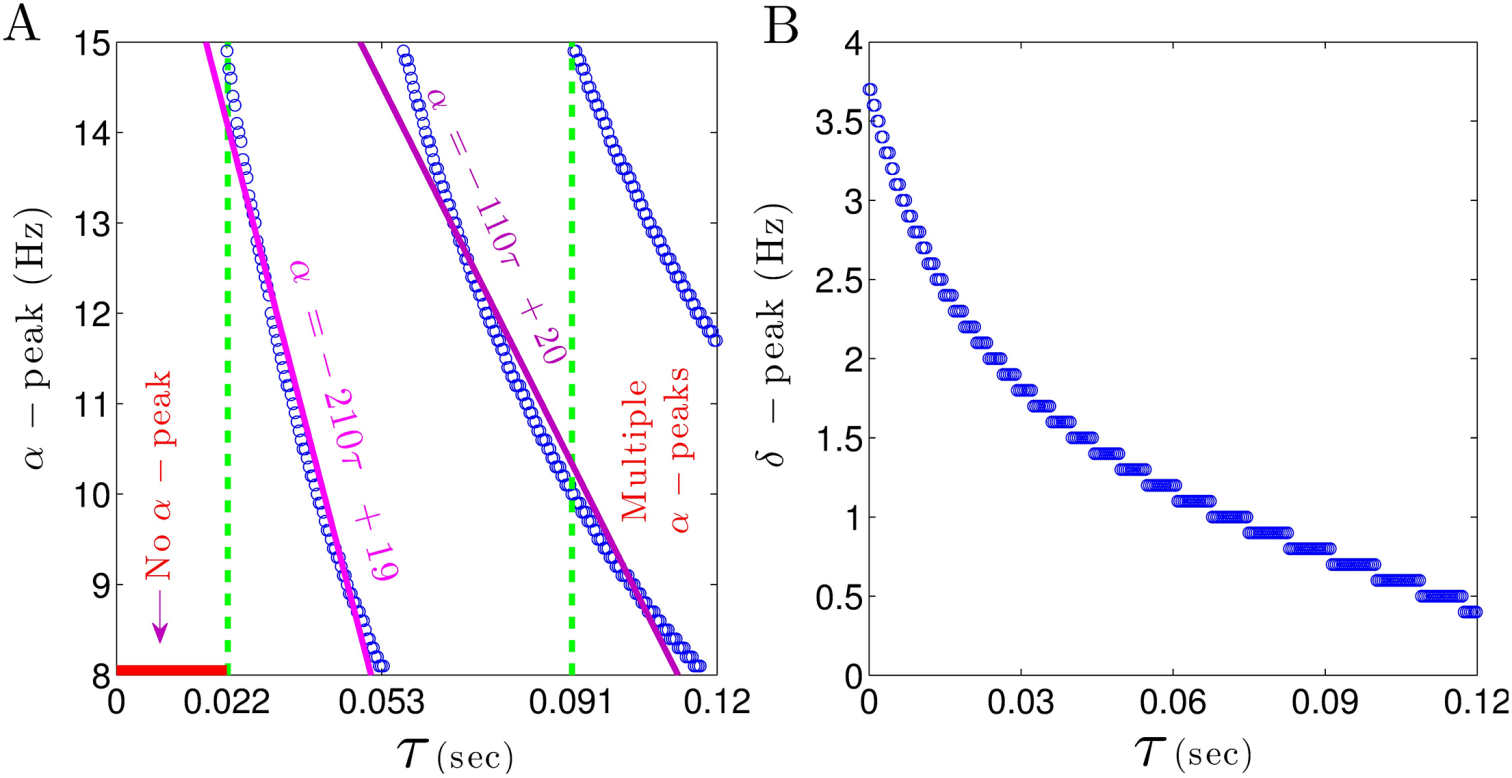
Spectral power peaks in the *δ*– and *α*–frequency range depend differently on the CTC delay. (A) For *τ* < 0.022 sec no peak in *α*–range can be observed (marked by a horizontal red line), whereas for *τ* ≥ 0.022 sec the *α*–power frequency decreases rapidly. (B) The peak frequency in the *δ*–frequency range decreases monotonically with increasing CTC delay *τ*. In all panels, unchanged parameters are taken from Table 1.

In order to take a closer look at the relation between *δ*– and *α*–power peaks and CTC time delay *τ*, the change in frequency of these peaks with respect to the CTC delay is illustrated in Fig 8. For small CTC delays (*τ* < 0.022 sec) EEG power spectra do not exhibit any peaks in *α*–frequency range (marked by a horizontal red line in Fig 8A). Moreover the peak frequency in the *α*–band abruptly decreases from 15 Hz to 8 Hz, when the CTC delay increases from 0.022 sec to 0.053 sec. A steep line fit to the *α*–peak frequency modulation (purple line in Fig 8A) illustrates the high sensitivity of the peak frequency to the CTC delay. Note that for larger delays, the frequency decreases not that fast with increasing CTC delay. Moreover, for *τ* > 0.091 sec, more than one peak in the *α*–range occur.

Conversely, the *δ*–power peak is very less sensitive to the CTC delay (Fig 8B). By increasing *τ*, the *δ*– peak frequency decreases gradually from 4 Hz to 0.5 Hz.

### Comparison to experimental EEG power spectrum

**Fig 9:**
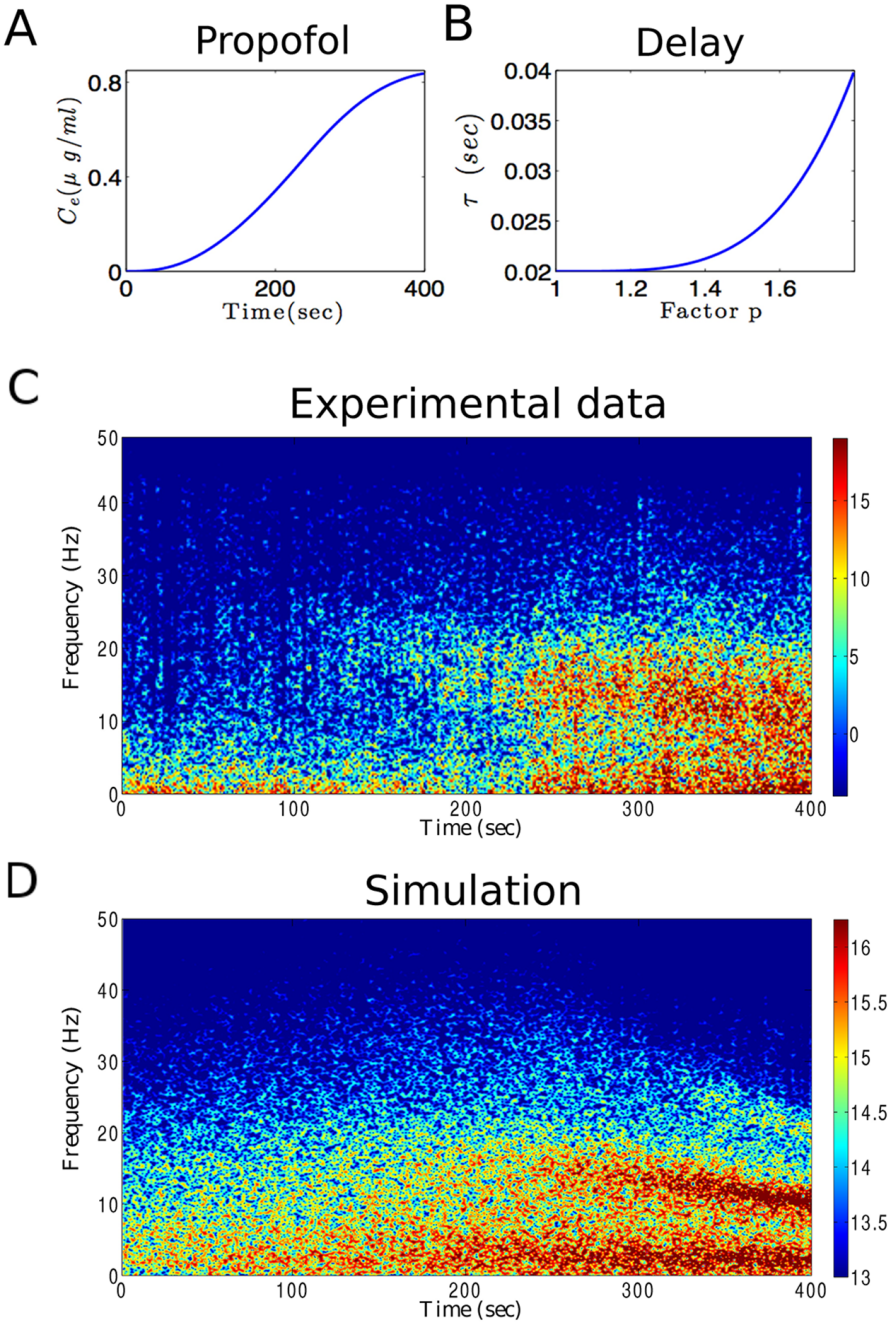
Experimental and theoretical spectrogram during propofol-induced anesthesia. (A) Effect site concentration of propofol (*C*_*e*_) as calculated from population based pharmacokinetic modelling (see e.g. [74, 75]) with respect to administration time. (B) To reproduce the shift in the dominant spectral power frequency, it is assumed that the CTC delay *τ* increases with propofol concentrations according to Eq (17). (C) The spectrogram of frontal EEG power observed in a single subject while the propofol concentration increases. (D) The spectrogram of simulation results based on the model.

By virtue of the insights into the effects of synaptic and feedback time scales, now we are able to develop a model of anesthetic action explaining the spectral power of experimental EEG subject to the propofol concentration. Fig 9 presents experimental EEG measured at a frontal electrode obtained during general anesthesia with propofol in a single subject. During the experiment, the blood concentration of propofol is increased in time (Fig 9A) leading to a gradual change of power spectrum in time (Fig 9C). Increasing propofol concentration leads to an increase in *δ*– and *α*–power activities. Moreover, we observe that spectral power increases at about 20 Hz after 120s whose spectral peak enhances and decreases in frequency in time down to 12 Hz after 400s. This emergent power peak is known as *beta buzz* and occurs in some patients under propofol anesthesia (see S1 Fig). It is a precursor of the patients loss of consciousness [9].

In order to explain and reproduce both the increase in *δ*– and *α*–activity and the beta buzz activity pattern, we re-consider the previous results on the impact of synaptic time scales and the CTC delay on the model power spectrum. Increasing levels of the anesthetic propofol decreases *β*_*i*_ and hence varies slightly the frequency of the *δ*– and *α*–power peaks, cf. Fig 2 in accordance to previous own studies [4, 13]. However, the modification of synaptic time scales does not yield a decrease of the *α*–peak from large to small frequencies as observed in the experimental data shown in Fig 9C. Consequently, another anesthetic action mechanism is necessary to explain the data.

Figs 6–8 reveal that changing the CTC delay *τ* with the propofol concentration may explain the emergence and behavior of the beta-buzz activity pattern. To achieve this effect, we presume that the CTC feedback delay increases while increasing the propofol concentration (see Fig 9B). Here, it is assumed that the CTC delay *τ* increases from 0.02 sec to 0.04 sec as the propofol concentration parameter *p* increases from *p* = 1 to *p* = 1.8 by

$$\begin{array}{c} \tau \left(p\right)=\tau_{0}+m{\left(p-1\right)}^{n}\;, \end{array}$$ (17)

where *τ*_0_ = 0.02 sec, and *n* = 4. The relation between CTC delay *τ* and the factor p is chosen in such a way that the value of *p* = 1.8 corresponds to *τ* = 0.04 sec leading to *m* = (0.04sec − 0.02sec)/(1.8 − 1)^*n*^ = 0.0488 sec. This choice leads to a gradual decrease in the spectral power peak, from around 20 Hz to 10 Hz, as observed in experiments. Fig 9D shows the simulation result (cf. section Theoretical power spectrum) based on the proposed model considering the propofol effects on inhibitory synapses as well as the time delay transmission. It can be observed that the EEG spectral power in *δ*– and *α*–frequency ranges enhances by increasing the propofol concentrations. Furthermore, in a close agreement with experimental observations, the dominant peak frequency in the thalamo-cortical oscillations decreases gradually in response to incremental increase in propofol concentration. As the factor *p* increases, the spectral peak frequency approximately shifts from 20 Hz to 10 Hz.

**Fig 10:**
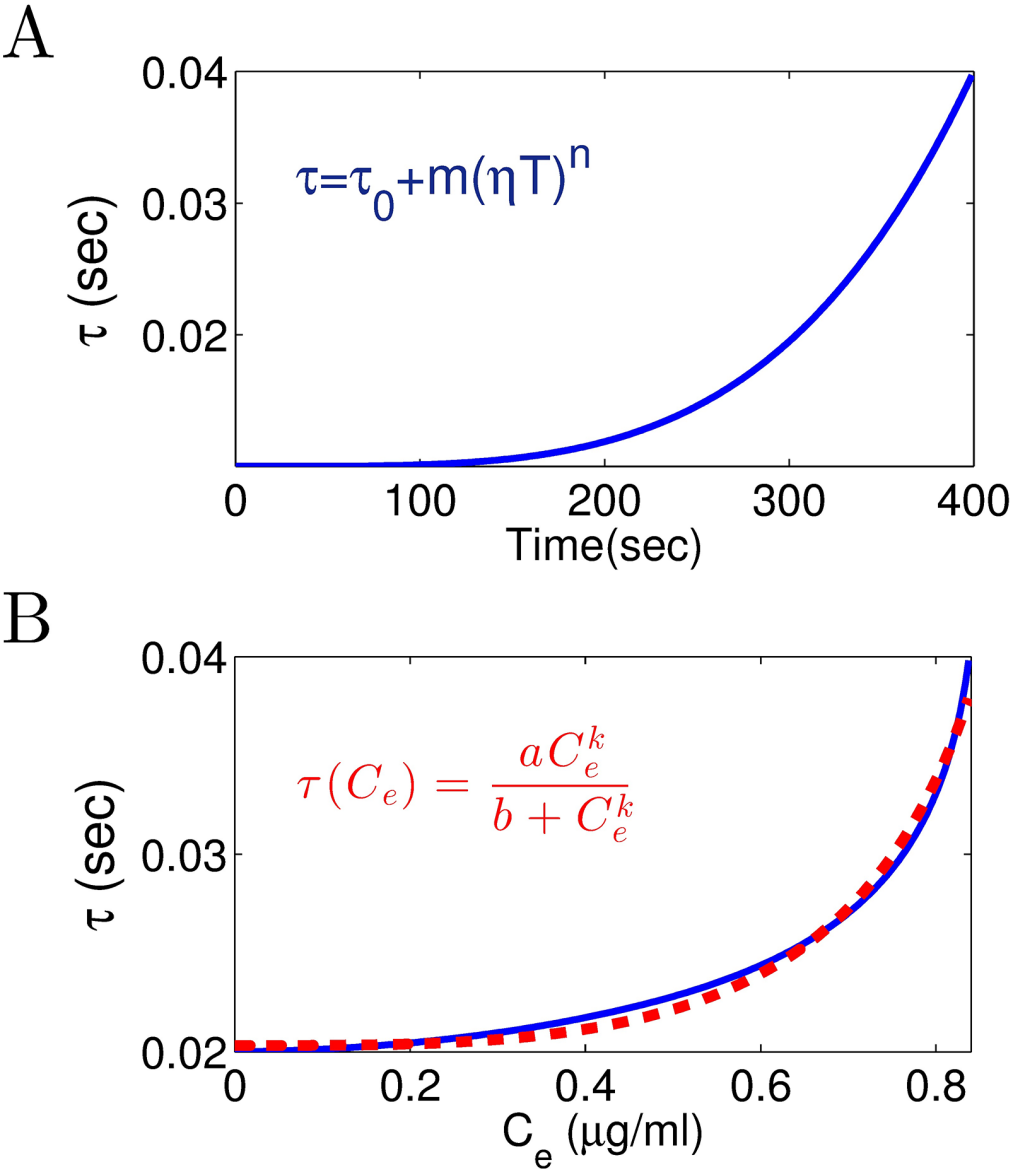
(A) According to Eq (18), the time delay *τ* is plotted with the respect to administration time (*T*). Note that inserting *p*(*T*) = *ηT* + 1 into Eq (17) gives the relation Eq (18). (B) From panels A and Fig 9A, time delay *τ* is plotted with the respect to propofol concentration *C*_*e*_ (blue line). The function Eq (19) is fitted to the extracted curve (dashed red line).

On the basis of these results, we estimate how the CTC delay *τ* varies with the blood plasma concentration of propofol. Please re-call that the delay *τ* is assumed to increase with increasing factor *p* according to Eq (17). Assuming that *p* increases linearly with increasing administration time, *p*(*T*) = *ηT* + 1 with *η* = (1.8 − 1)/(400sec − 0sec) = 0.002 Hz. From these equations, the value of delay *τ* depends on the administration time by

$$\begin{array}{c} \tau \left(T\right)=\tau_{0}+m{\left(\eta T\right)}^{n}\;, \end{array}$$ (18)

see Fig 10A. Now by considering the values of *C*_*e*_ with respect to *T* and *τ* subjected to *T*, we can estimate how the time delay *τ* varies with the propofol concentration (see blue line in Fig 10B). To this end, fitting the function

$$\begin{array}{c} \tau \left(C_{e}\right)=\frac{aC_{e}^{k}}{b+C_{e}^{k}} \end{array}$$ (19)

to the experimental data yields the wanted relationship between the CTC delay transmission and the propofol concentration. In Fig 10B, the dashed red line illustrates the fitted function with *a* = 0.0203, *b* = −0.8411, and *k* = −3.3492. Summarizing, a good estimate of the parameters in Eq (17) allows to fit the relationship between the thalamo-cortical delay and the propofol plasma concentration.

### Constrains on system stability

Since the power spectrum analysis is valid only if the resting state of the system is stable, we investigate the conditions which guarantee that the thalamo-cortical system exhibits stable oscillations. To derive the stability conditions analytically, we need to simplify the model under study. In the first step, we neglect the cortical inhibitory neurons (I) in the thalamo-cortical system (cf. Fig 1). Several lines of evidence indicate that the thalamus is an important propofol target-site and thalamic neuronal circuits play a critical role in contributing to propofol general anesthesia [76–78]. In our previous work [13], we have shown that by neglecting the cortical inhibitory population, thalamic inhibition is still adequate to reproduce observed changes in EEG rhythms within *δ*– and *α*–activity bands during propofol-induced anesthesia. In other words, thalamic inhibition balances cortical excitation as cortical inhibition does. Secondly, as observed in Figs 2 and 4, the rise time constants of the response functions for excitatory and inhibitory synapses have no effect on EEG power peaks in *δ*– and *α*frequency ranges. It is also widely accepted that anesthetic agent propofol prolongs the temporal decay phase of inhibitory synapses only while the inhibitory rise rate and the synaptic time constants of excitatory synapses remain unaffected [35]. According to these findings, in a reasonable approximation, we assume an instantaneous rise of the synaptic response function followed by an exponential decay phase i.e., *α*_*e*_, *α*_*i*_ → ∞. This approximation simplifies the second-order temporal operators ${\hat{L}}_{e,i}$ (cf. Eq (3)) to the first-order operators ${\hat{L}}_{k}=1+i\omega /\beta_{k}\;,k=e,i$. Applying these simplifications, the power spectrum of EEG is

$$\begin{array}{c} P_{E}\left(\omega \right)=2\kappa \sqrt{2\pi }{\left|{\tilde{G}}_{1,5}\left(\omega \right)\right|}^{2}, \end{array}$$ (20)

where

$$\begin{array}{c} {\tilde{G}}_{1,5}\left(\omega \right)=\frac{-K_{1}{\hat{L}}_{i}e^{-i\omega \tau_{TC}}}{{\hat{L}}_{e}\left({\hat{L}}_{e}{\hat{L}}_{i}+C_{2}\right)+e^{-i\omega (\tau_{CT}+\tau_{TC})}\left(C_{3}-C_{1}{\hat{L}}_{i}\right)}, \end{array}$$ (21)

with *C*_1_ = *K*_2_*K*_6_, *C*_2_ = *f*_*T*_(*p*)*K*_7_*K*_9_ and *C*_3_ = *f*_*T*_(*p*)*K*_2_*K*_7_*K*_8_ (see S2 Appendix for definition of *K*_*i*_). Thus, the characteristic equation of the system under study (the denominator of ${\tilde{G}}_{1,5}$) reduces to a third-order equation, which is analytically more tractable. Our simulations demonstrate that the mentioned simplifications have no significant effect on the EEG spectral power in *δ*– and *α*–frequency ranges (not shown). It is important to note that since $|e^{-i\omega \tau_{TC}}|=1$, the absolute value of numerator of ${\tilde{G}}_{1,5}$ does not depend to *τ*_*TC*_, and thus the spectral power given by Eq (20) is just affected by the CTC delay *τ* = *τ*_*CT*_ + *τ*_*TC*_ only.

In the following, we employ the stability criterion from section Stability analysis of the linear model to derive analytical conditions for the stability of the system. In this method, we first probe the conditions under which in the absence of time delay (*τ* = 0) the system is stable. Then, by increasing the delay value, we investigate whether or not there exists a critical delay for which the system becomes unstable (the introduction of delay yields a bifurcation).

In S4 Appendix), we have shown that the following conditions guarantee the stability of the system in the absence of delay, and the introduction of a time delay can not cause a bifurcation and hence, for *τ* ≥ 0, the reduced thalamo-cortical model exhibits stable oscillations:

$$\begin{array}{ccc} \beta_{i}\left(C_{2}+2\right)+\beta_{e}\left(1-C_{1}\right) & > & 0,\;\;\;(\text{Condition}\;\text{I})\\ C_{3}+C_{2}-C_{1}+1 & > & 0,\;\;\;(\text{Condition}\;\text{II})\\ \left(2\beta_{e}+\beta_{i}\right)\left(\frac{C_{2}+2}{\beta_{e}}+\frac{1-C_{1}}{\beta_{i}}\right)-\left(C_{3}+C_{2}-C_{1}+1\right) & > & 0,\;\;\;(\text{Condition}\;\text{III})\\ {\left(\beta_{e}^{2}\beta_{i}\right)}^{2}\left({\left(C_{2}+1\right)}^{2}-{\left(C_{3}-C_{1}\right)}^{2}\right) & > & 0,\;\;\;(\text{Condition}\;\text{IV})\\ \Delta <0,\;\;or\;\;if\;\;\Delta \geq 0\;then\;\xi_{1},\xi_{2} & > & 0.\;\;\;\;(\text{Condition}\;\text{V}) \end{array}$$

**Fig 11:**
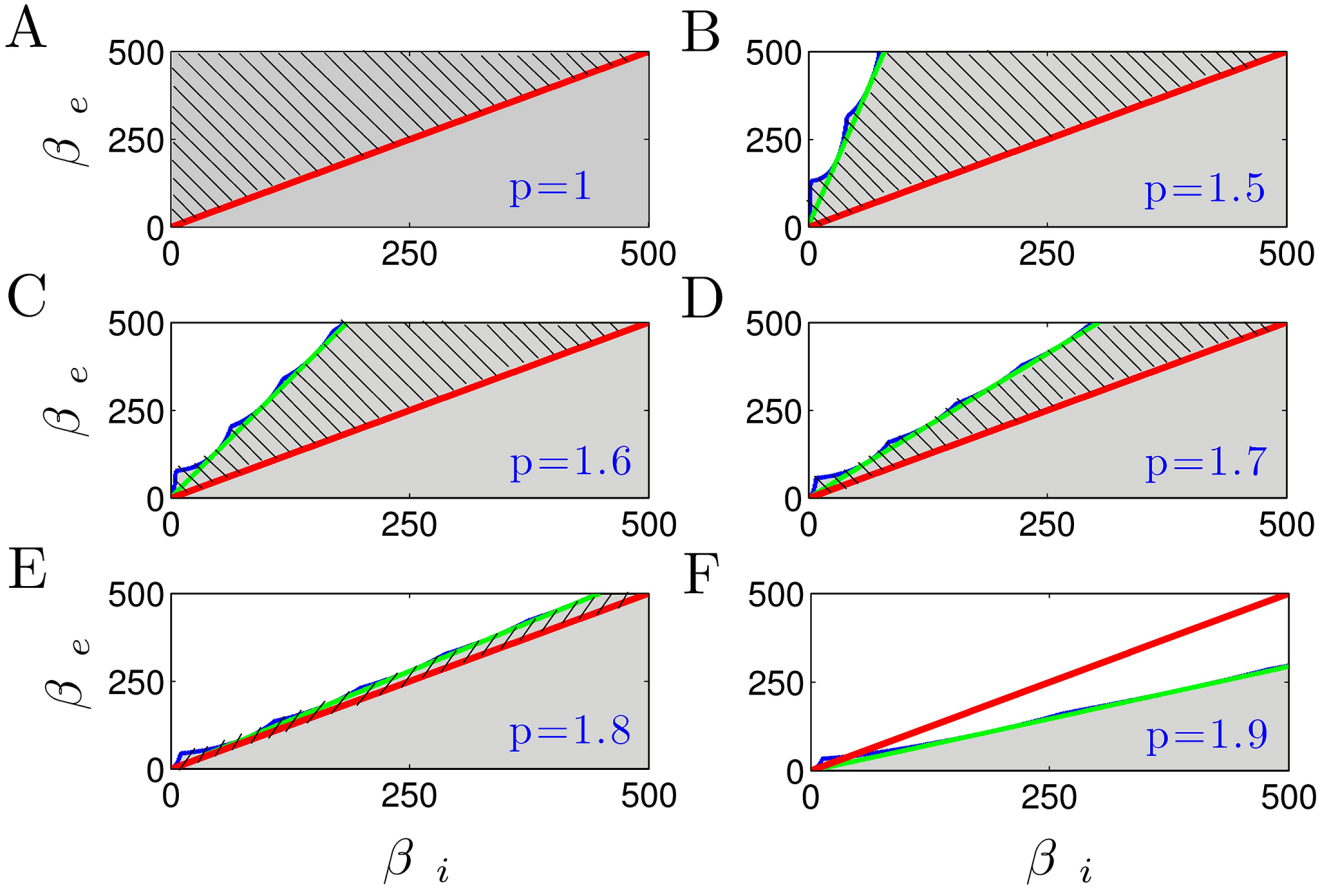
The stability regions with respect to the excitatory and inhibitory synaptic decay rates of the reduced thalamo-cortical model. The shaded (unshaded) areas represent the parameter regions, where the system exhibits stable (unstable) oscillations. The green lines show the thresholds of parameter zones that are consistent with the multiple constraints (I)-(V), whereas the stability thresholds obtained numerically are encoded in blue lines. The area above the red line indicates the physiologic limit defined by *β*_*e*_ > *β*_*i*_. Hence, the shaded region with hatching represents the physiological range of parameter spaces, for which the system is stable. Note that here the CTC delay has a constant value of *τ* = 0.04 sec.

For different anesthetic levels, we consider the parameter space defined by decay rates of excitatory and inhibitory synapses (*β*_*e*_, *β*_*i*_). Fig 11 shows the parameter regime (shaded areas) where the system exhibit stable oscillations. The stability thresholds of the parameter regime that satisfies the stability conditions (I)-(V) are denoted by green lines, whereas those obtained numerically are encoded in blue lines. Firstly one observes a very close accordance of the analytical and the numerical stability borders. Secondly, it can be seen that increasing anesthetic concentration (factor *p*) shrinks the stability regions. In other words, the increase in inhibitory decay rate *β*_*i*_ decreases the stability of the system.

It is important to note that the response functions for the inhibitory synapses exhibit a larger synaptic time constants than the excitatory synapses, i.e., *β*_*e*_ > *β*_*i*_. This physiological limit is equivalent to the parameter region above the line *β*_*e*_ = *β*_*i*_ (red line). Thus, the hatched shaded areas in the plot represent the physiologically possible combinations of *β*_*e*_ and *β*_*i*_, where the parameters are consistent with all the stability constraints simultaneously. It can be observed that a high anesthetic level (e.g., *p* = 1.8) yields a small physiological region of the parameter space only, where the system exhibit stable oscillations (see Fig 11E). When *p* = 1.9, for the physiological parameter zone *β*_*e*_ > *β*_*i*_, there is no region in which the parameters satisfy the both physiological limit and the multiple stability constrains (see Fig 11F).

## Discussion

### Anesthetic action on synapses affects spectral power but does not induce beta-buzz

The anesthetic propofol is known to affect the charge transfer and the response time scales of synapses and tunes spectral power in the *δ*– and *α*–frequency bands. Figs 2 and 3 clearly demonstrate that a decrease of *β*_*i*_ by increasing the propofol concentration modifies the frequency and magnitude of the *δ*– and *α*–peak in good accordance to previous work [3, 13]. In addition, the excitatory and inhibitory rise rates *α*_*e*,*i*_ poorly affect the spectral power in the *δ*– and *α*–frequency range. This is in line with a previous own study [13] reproducing experimental power spectra while neglecting the rise phase of synaptic response activities. This possible neglect of the synaptic rise phase makes it possible to reduce the model to a well-reduced effective model whose dynamical features retain the major dynamical mechanisms while pointing out their importance. In spite of these anesthetic effects on synapses, the synaptic action does not allow explain the dramatic frequency shift of the beta-buzz shown in Fig 9.

### Anesthetic action on cortico-thalamo-cortical (CTC) delay affects the *δ*– and *α*–power and may induce the beta-buzz

Fig 6 shows that the cortico-thalamic and the thalamo-cortical transmission delay plays a critical role in *α*–power modulation. For increases of the sum of both delays the peak frequency of *α*–power decreases as seen in Figs 7 and 8A. This evolution of spectral power resembles well the evolution observed experimentally as the beta-buzz observed in Fig 9C [8, 9]. By virtue of this similarity, we propose the model Eq (19) introducing a relationship between the CTC delay and the anesthetic blood concentration. The temporal evolution of the spectral power of the resulting model activity (Fig 9C) resembles well the experimental data.

Several previous modeling studies have explained the *α*–rhythm by the delayed thalamo-cortical feedback [15, 18, 31, 37, 79, 80]. These hypotheses point out that the *α*–activity results from an interaction between two brain structures rather than being generated in single area [81, 82]. Other studies argue that *α*– and *δ*– rhythms can be explained by cortical interactions only [60, 83]. Moreover, there is experimental evidence that *α*–activity has a thalamic origin [84] that is facilitated by thalamic gap-junctions [85–87] or extra-synaptic GABA-receptors [4].

Taking together the myriade of previous studies, it is important to note that there is no ‘either-or’ in models but different successful models just indicate various mechanisms that might contribute to the experimental findings. The fact that anesthetic action induces very similar behavior in all subjects, such as sedation and loss of consciousness, while the neural structure of different subjects are different and, in addition, this structure changes on a time scale of days and weeks [88] indicates a fundamental underlying mechanism of general anesthesia common to all subjects. By virtue of the diversity of subjects and plastic neural structures, we argue that this mechanism is rather independent on the fine network scale of the brain. A good candidate for such a mechanism is the interaction of excitation and inhibition on a global network scale, which is implemented in both successful cortico-thalamic feedback models and cortical models. In fact, a recent model study [13] has demonstrated that the level of excitation and inhibition both in a detailed complex and in a well-reduced global network of neural populations is sufficient to explain the generation of EEG spectral features under anesthesia. Our model considers the effect of anesthetics on the level of excitation and inhibition as well and captures well the EEG signals (see S2 Fig).

### CTC delay depends on the anesthetic concentration

The novel relationship Eq (19) results from the ability of the model to reproduce the beta-buzz by an anesthetic-dependent delay between cortical and thalamic structures. This raises the question which physiological elements in the corresponding neural connection are affected by the anesthetics.

To understand this, one should recall that in the brain the direct feedback connections sketched in Fig 1 are complemented by indirect connections by areas close to the thalamus such as the globus pallidus and the striatum and, more distant, via e.g. the hypothalamus, the basal forebrain and the ventral tegmental area [1]. Synaptic time scales of excitatory and inhibitory synapses in these neural structures are affected by anesthetics. The prolongation of the corresponding time scales modify effectively the delay between thalamus and cortex yielding its effective prolongation with increasing anesthetic concentration. This picture fits to the model presented since this actually represents a simplified, rather abstract, dynamical model capturing the major dynamical features of the underlying neural dynamical interaction on several scales [42].

A closer look at the model proposed reveals that, in fact, the functions Eq (2) describe the mean post-synaptic responses to incoming action potentials considering a single time scale for the synaptic decay phase. However, it is well-known from experiments, that there exists several decay phases in synaptic response with a prolonged total decay phase. This finding indicates a mechanism of desensitization [32]. The question whether anesthetics enhance desensitization is under discussion, cf. [89]. However, there is growing evidence that anesthetics prolong the decay phase of synaptic responses for several general anesthetics [53] including propofol [33, 90]. Our proposed model considers a single decay phase only and hence does not take into account the synaptic response delay induced by desensitization. The CTC model delay captures this additional delay. Consequently increasing the anesthetic concentration yields an enhancement of desensitization and hence a prolonged in the synaptic response in connections between cortex and thalamus. Consequently desensitization effects may explain the suggested prolongation of the CTC delay by increasing propofol concentration.

Furthermore, it is important to mention that there is large variability in the data between patients (as can be seen in S1 Fig) and there is no single mechanism to explain it. Our work provides one possible mechanism among many unknown underlying mechanisms that contribute to the beta-buzz phenomenon.

### Propofol destabilizes the resting state of wakefulness

The suggested model allows a close reproduction of spectral features of experimental EEG. It considers dynamical interactions between cortical and thalamic neural populations evolving about a time-independent resting state. The EEG is assumed to result from small deviations about this resting state. Since this assumption implies an asymptotically stable resting state for all parameters under study, it is mandatory to ensure the stability of the resting state. Section Constrains on system stability gives the corresponding analytical conditions. Fig 11 clearly demonstrates that increasing the propofol concentration reduces the domain of parameters for which the resting state is stable. Hence the general anesthetic propofol destabilizes the resting state at which the subject was un-anesthetized. This destabilization is in full line with previous models explaining the loss of consciousness by a phase transition [58, 91]. Experimental evidence of neural inertia [92] also supports the hypothesis of phase transition-like change of the brains state. Neural inertia involves a genetics-based switch between consciousness and anesthetic-induced unconsciousness [93] and the transition between consciousness and unresponsiveness to external commands is preceded by the systems movement towards its stability threshold.

The present considers resting state EEG as it may occur in hospital practice, e.g. during surgery. A further major interest in research of general anesthesia is the understanding how the brain blocks external commands corresponding to a loss of consciousness, i.e. a blockage of neural information processing and information sharing. It has been shown in several previous studies that anesthetics may diminish [10, 94] or augment evoked neural responses [95] to experimentally induced stimulations. Our current work does not consider such induced activity while it will be the focus of forthcoming studies taking into account the anesthetic action on the delayed neural activity transmission between cortex and thalamus.

## Supporting information

S1 Fig Experimental EEG spectrogram during propofol-induced anesthesia. The spectrogram of frontal EEG power observed in eight subjects while the propofol concentration increases. The blood plasma concentration of propofol with respect to administration time was shown in Fig 9A.
(TIF)

S2 Fig Comparisons between experimental and simulated EEG time-series in the baseline and anesthesia conditions. Panels (A) and (B) illustrate the recorded EEG time-series in awake (blue) and anesthesia (red) conditions, respectively. Panels (C) and (D) show the corresponding simulations. Both real and simulated data show that increasing propofol concentration changes the EEG from high frequency-low amplitude signals (corresponds to awake condition, with *p* = 1) to low frequency-high amplitude signals (corresponds to anesthesia condition, with *p* > 1).
(TIF)

S1 Appendix Theoretical power spectrum. (PDF)

S2 Appendix Theoretical EEG power spectrum. (PDF)

S3 Appendix Finding the system characteristic roots. (PDF)

S4 Appendix Constrains on system stability. (PDF)
