# Supplementary figures and images for: Anesthetic action on the transmission delay between cortex and thalamus explains the beta-buzz observed under propofol anesthesia
Source: PLoS One. 2017 Jun 16;12(6):e0179286. doi: 10.1371/journal.pone.0179286 (PMC5473556; doi:10.1371/journal.pone.0179286)

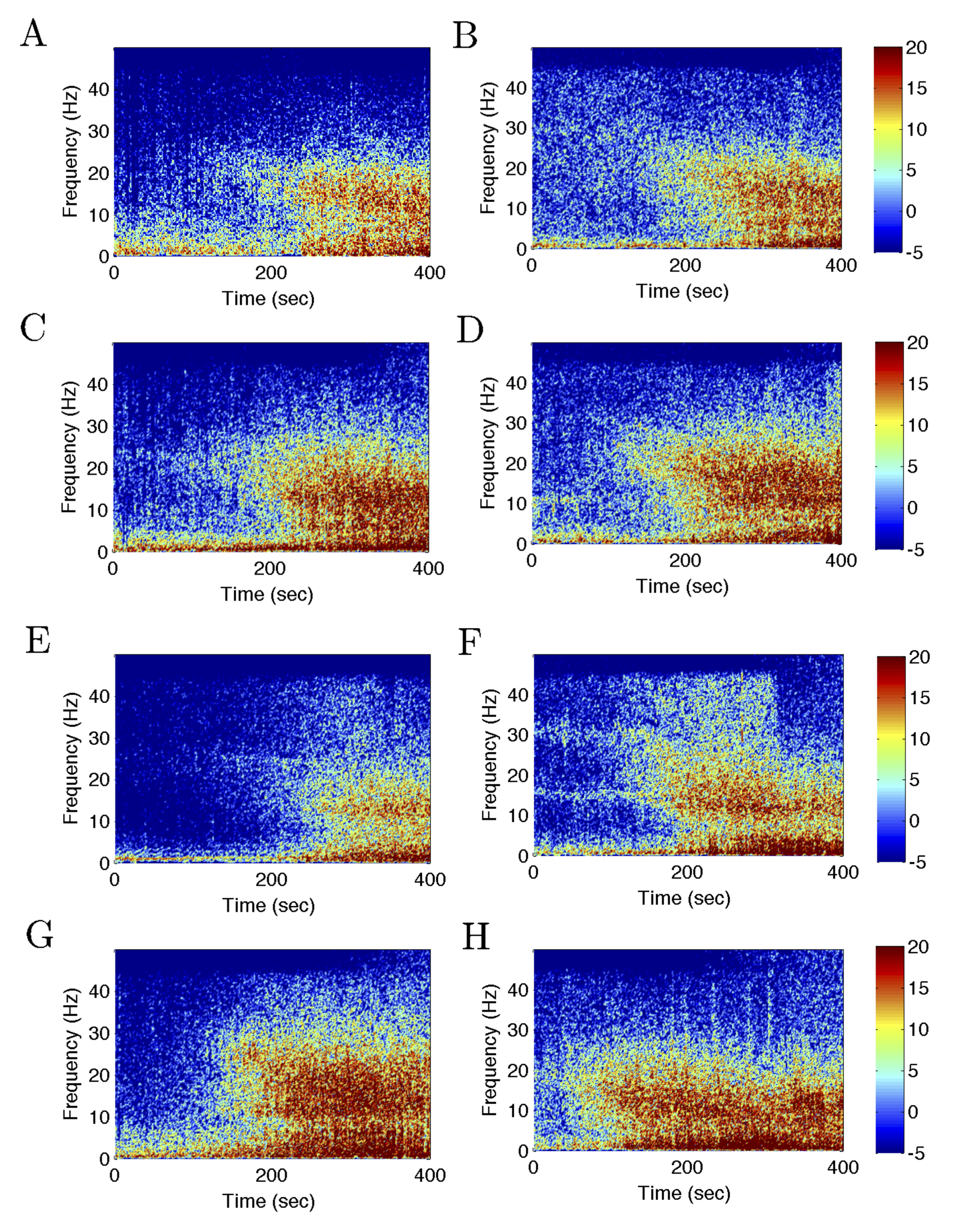

Supplement: S1 Fig — The spectrogram of frontal EEG power observed in eight subjects while the propofol concentration increases. The blood plasma concentration of propofol with respect to administration time was shown in Fig 9A. (TIF) [file pone.0179286.s001.tif]

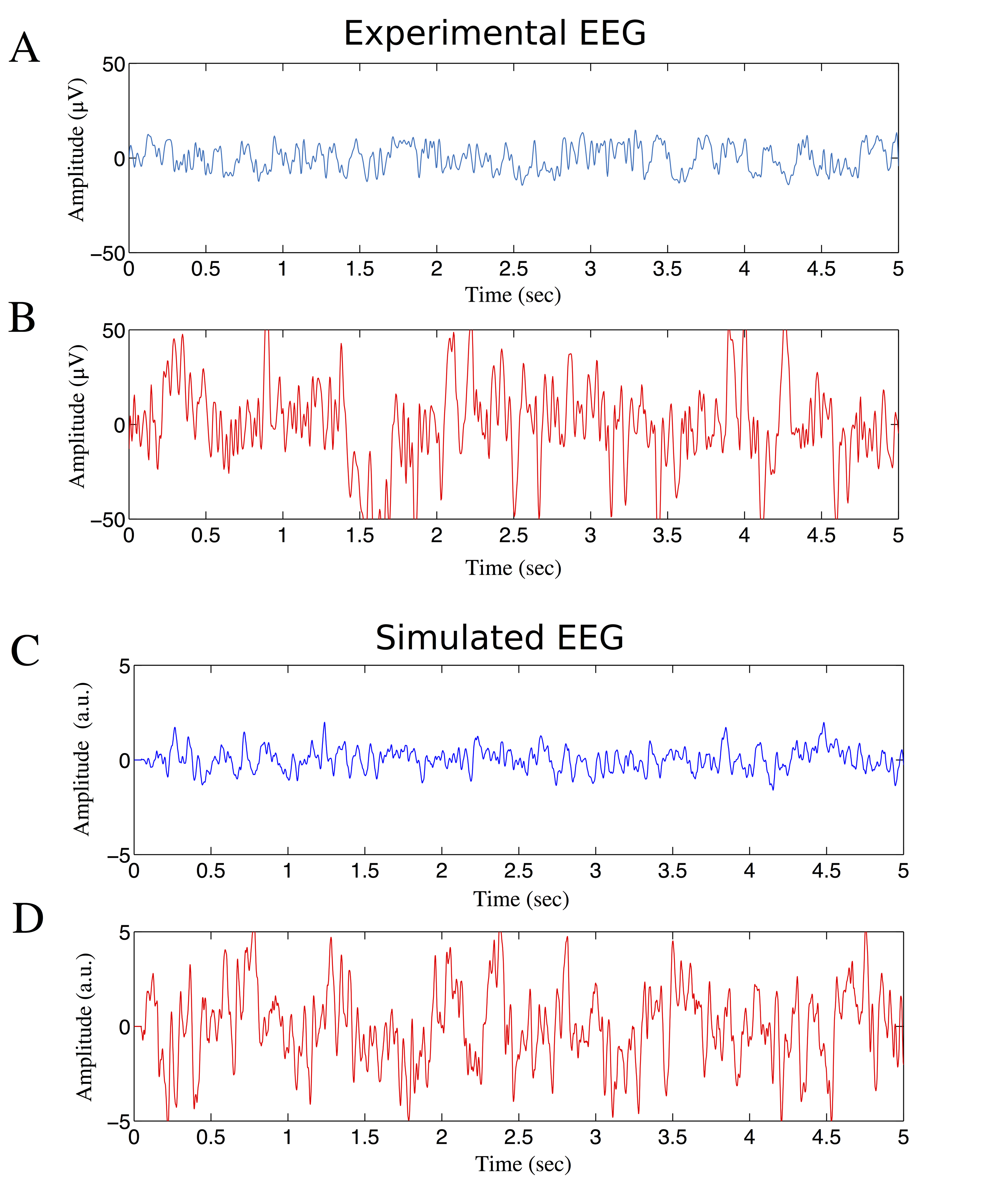

Supplement: S2 Fig — Panels (A) and (B) illustrate the recorded EEG time-series in awake (blue) and anesthesia (red) conditions, respectively. Panels (C) and (D) show the corresponding simulations. Both real and simulated data show that increasing propofol concentration changes the EEG from high frequency-low amplitude signals (corresponds to awake condition, with p = 1) to low frequency-high amplitude signals (corresponds to anesthesia condition, with p > 1). (TIF) [file pone.0179286.s002.tif]
