# Supplementary material for: Anesthetic action on the transmission delay between cortex and thalamus explains the beta-buzz observed under propofol anesthesia
Source: PLoS One. 2017 Jun 16;12(6):e0179286. doi: 10.1371/journal.pone.0179286 (PMC5473556; doi:10.1371/journal.pone.0179286)
Supplement: S1 Appendix — (PDF) [file pone.0179286.s003.pdf]

S1 Appendix. Theoretical power spectrum.

The solution of

$$\hat{\boldsymbol{L}}(\partial/\partial t)\boldsymbol{Y}(t) = \boldsymbol{A}\boldsymbol{Y}(t) + \boldsymbol{B}\boldsymbol{Y}(t-\tau_{TC}) + \boldsymbol{C}\boldsymbol{Y}(t-\tau_{CT}) + \boldsymbol{\xi}(t), \quad (1)$$

for $t \to \infty$ is

$$\boldsymbol{Y}(t) = \int_{-\infty}^{\infty} \boldsymbol{G}(t-t')\boldsymbol{\xi}(t')dt', \quad (2)$$

with the matrix Green's function $\boldsymbol{G} \in \mathbb{R}^{N\times N}$. Substituting Eq (2) into Eq (1) leads to

$$\hat{\boldsymbol{L}}(\partial/\partial t)\boldsymbol{G}(t) = \boldsymbol{A}\boldsymbol{G}(t) + \boldsymbol{B}\boldsymbol{G}(t-\tau_{TC}) + \boldsymbol{C}\boldsymbol{G}(t-\tau_{CT}) + \boldsymbol{1}\delta(t), \quad (3)$$

with the unitary matrix $\boldsymbol{1} \in \mathbb{R}^{N\times N}$. Applying the Fourier transform

$$\boldsymbol{G}(t) = \frac{1}{\sqrt{2\pi}} \int_{-\infty}^{\infty} \tilde{\boldsymbol{G}}(\omega)e^{i\omega t}d\omega, \quad (4)$$

yields

$$\tilde{\boldsymbol{G}}(\omega) = \frac{1}{\sqrt{2\pi}} \left[\boldsymbol{L}(\omega) - \boldsymbol{A} - \boldsymbol{B}e^{-i\omega\tau_{TC}} - \boldsymbol{C}e^{-i\omega\tau_{CT}}\right]^{-1}. \quad (5)$$

The power spectral density matrix $\boldsymbol{P}(\omega)$ of $\boldsymbol{Y}(t)$ is the Fourier transform of the auto-correlation function matrix $\langle \boldsymbol{Y}(t)^t\boldsymbol{Y}(t-T)\rangle$ (Wiener-Khinchine Theorem) leading to

$$\boldsymbol{P}(\omega) = 2\kappa\sqrt{2\pi}\tilde{\boldsymbol{G}}(\omega)\tilde{\boldsymbol{G}}^{\top}(-\omega).$$

Finally, by virtue of the specific choice of external input to the j-th element of the activity variable, the power spectrum of i-th element just depends on one matrix component of the matrix Green's function by

$$P_i(\omega) = 2\kappa\sqrt{2\pi}\left|\tilde{G}_{i,j}(\omega)\right|^2. \quad (6)$$
