# Supplementary material for: Anesthetic action on the transmission delay between cortex and thalamus explains the beta-buzz observed under propofol anesthesia
Source: PLoS One. 2017 Jun 16;12(6):e0179286. doi: 10.1371/journal.pone.0179286 (PMC5473556; doi:10.1371/journal.pone.0179286)
Supplement: S2 Appendix — (PDF) [file pone.0179286.s004.pdf]

S2 Appendix. Theoretical EEG power spectrum.

Under the assumption of the spatial homogeneity, the mean excitatory and inhibitory postsynaptic potentials $V_a^{e,i}$ for neural populations $a \in \{E, I, R, S\}$ shown in Fig 1 obey the following set of coupled SDDEs

$$\begin{aligned}
\hat{L}_e V_E^e(t) &= K_{EE} S_C[V_E^e(t) - V_E^i(t)] + \\
&\quad K_{ES} S_T[V_S^e(t - \tau_{CT}) - V_S^i(t - \tau_{CT})], \\
\hat{L}_i V_E^i(t) &= f_C(p) K_{EI} S_C[V_I^e(t) - V_I^i(t)], \\
\hat{L}_e V_I^e(t) &= K_{IE} S_C[V_E^e(t) - V_E^i(t)], \\
\hat{L}_i V_I^i(t) &= K_{II} S_C[V_I^e(t) - V_I^i(t)], \\
\hat{L}_e V_S^e(t) &= K_{SE} S_C[V_E^e(t - \tau_{TC}) - V_E^i(t - \tau_{TC})] + I(t), \\
\hat{L}_i V_S^i(t) &= f_T(p) K_{SR} S_T[V_R^e(t)], \\
\hat{L}_e V_R^e(t) &= K_{RE} S_C[V_E^e(t - \tau_T) - V_E^i(t - \tau_{TC})] + \\
&\quad K_{RS} S_T[V_S^e(t) - V_S^i(t)].
\end{aligned} \tag{1}$$

When the system is driven by a constant, uniform mean stimulus level $I(t) = I_0$, the spatially-homogeneous resting state of Eqs (1) can be obtained by setting all of the derivatives to zero, i.e., $dV_a^{e,i}/dt = 0$, for $a \in \{E, I, S, R\}$ leading to

$$\begin{aligned}
V_E^{*e} &= K_{EE} S_C[V_E^{*e} - V_E^{*i}] + K_{ES} S_T[V_S^{*e} - V_S^{*i}], \\
V_E^{*i} &= f_C(p) K_{EI} S_C[V_I^{*e} - V_I^{*i}], \\
V_I^{*e} &= K_{IE} S_C[V_E^{*e} - V_E^{*i}], \\
V_I^{*i} &= K_{II} S_C[V_I^{*e} - V_I^{*i}], \\
V_S^{*e} &= K_{SE} S_C[V_E^{*e} - V_E^{*i}] + I_0, \\
V_S^{*i} &= f_T(p) K_{SR} S_T[V_R^{*e}], \\
V_R^{*e} &= K_{RE} S_C[V_E^{*e} - V_E^{*i}] + K_{RS} S_T[V_S^{*e} - V_S^{*i}].
\end{aligned} \tag{2}$$

Linearizing Eqs (1) at the resting state $\boldsymbol{X}_0 = (V_E^{*e}, V_E^{*i}, V_I^{*e}, V_I^{*i}, V_S^{*e}, V_S^{*i}, V_R^{*e})^\top$ yields

$$\hat{\boldsymbol{L}}(\partial/\partial t)\boldsymbol{Y}(t) = \boldsymbol{A}\boldsymbol{Y}(t) + \boldsymbol{B}\boldsymbol{Y}(t - \tau_{TC}) + \boldsymbol{C}\boldsymbol{Y}(t - \tau_{CT}) + \boldsymbol{\xi}(t). \tag{3}$$

where

$$\begin{aligned}
\boldsymbol{Y}(t) = (&V_E^e(t) - V_E^{*e}, V_E^i(t) - V_E^{*i}, V_I^e(t) - V_I^{*e}, V_I^i(t) - V_I^{*i}, \\
&V_S^e(t) - V_S^{*e}, V_S^i(t) - V_S^{*i}, V_R^e(t) - V_R^{*e})^\top,
\end{aligned}$$

$\boldsymbol{\xi}(t) = (0,0,0,0,\sqrt{2\kappa}\xi(t),0,0)^\top$, and the diagonal matrix $\hat{\boldsymbol{L}}(\partial/\partial t)$ with the entries $\hat{L}_{1,1} = \hat{L}_{3,3} = \hat{L}_{5,5} = \hat{L}_{7,7} = \hat{L}_e(\omega)$, and $\hat{L}_{2,2} = \hat{L}_{4,4} = \hat{L}_{6,6} = \hat{L}_i(\omega)$, and

$$\boldsymbol{A} = \begin{pmatrix} K_1 & -K_1 & 0 & 0 & 0 & 0 & 0 \\ 0 & 0 & f_C(p)K_3 & -f_C(p)K_3 & 0 & 0 & 0 \\ K_4 & -K_4 & 0 & 0 & 0 & 0 & 0 \\ 0 & 0 & K_5 & -K_5 & 0 & 0 & 0 \\ 0 & 0 & 0 & 0 & 0 & 0 & 0 \\ 0 & 0 & K_5 & -K_5 & 0 & 0 & f_T(p)K_7 \\ 0 & 0 & 0 & 0 & K_9 & -K_9 & 0 \end{pmatrix},$$

$$\boldsymbol{B} = \begin{pmatrix} 0 & 0 & 0 & 0 & 0 & 0 & 0 \\ 0 & 0 & 0 & 0 & 0 & 0 & 0 \\ 0 & 0 & 0 & 0 & 0 & 0 & 0 \\ 0 & 0 & 0 & 0 & 0 & 0 & 0 \\ K_6 & -K_6 & 0 & 0 & 0 & 0 & 0 \\ 0 & 0 & 0 & 0 & 0 & 0 & 0 \\ K_8 & -K_8 & 0 & 0 & 0 & 0 & 0 \end{pmatrix}, \boldsymbol{C} = \begin{pmatrix} 0 & 0 & 0 & 0 & K_2 & -K_2 & 0 \\ 0 & 0 & 0 & 0 & 0 & 0 & 0 \\ 0 & 0 & 0 & 0 & 0 & 0 & 0 \\ 0 & 0 & 0 & 0 & 0 & 0 & 0 \\ 0 & 0 & 0 & 0 & 0 & 0 & 0 \\ 0 & 0 & 0 & 0 & 0 & 0 & 0 \\ 0 & 0 & 0 & 0 & 0 & 0 & 0 \end{pmatrix},$$

with

$$\begin{aligned}
\hat{L}_e(\omega) &= \left(1+\frac{i\omega}{\alpha_e}\right)\left(1+\frac{i\omega}{\beta_e}\right), & \hat{L}_i(\omega) &= \left(1+\frac{i\omega}{\alpha_i}\right)\left(1+\frac{i\omega}{\beta_i}\right), \\
K_1 &= K_{EE}\frac{dS_C[V]}{dV}\,|_{V=(V_E^{*e}-V_E^{*i})}, & K_2 &= K_{ES}\frac{dS_T[V]}{dV}\,|_{V=(V_S^{*e}-V_S^{*i})}, \\
K_3 &= K_{EI}\frac{dS_C[V]}{dV}\,|_{V=(V_I^{*e}-V_I^{*i})}, & K_4 &= K_{IE}\frac{dS_C[V]}{dV}\,|_{V=(V_E^{*e}-V_E^{*i})}, \\
K_5 &= K_{II}\frac{dS_C[V]}{dV}\,|_{V=(V_I^{*e}-V_I^{*i})}, & K_6 &= K_{SE}\frac{dS_C[V]}{dV}\,|_{V=(V_E^{*e}-V_E^{*i})}, \\
K_7 &= K_{SR}\frac{dS_T[V]}{dV}\,|_{V=V_R^{*e}}, & K_8 &= K_{RE}\frac{dS_C[V]}{dV}\,|_{V=(V_E^{*e}-V_E^{*i})}, \\
K_9 &= K_{RS}\frac{dS_T[V]}{dV}\,|_{V=(V_S^{*e}-V_S^{*i})}\,.
\end{aligned}$$

Note that the constants $K_i,\ i = 1,\ldots,9$ are proportional to the nonlinear gain $dS(V)/dV$ computed at the resting state of the system.

If we assume that the EEG is generated by activity of cortical pyramidal cells V_E^e, and the external input is projected to the excitatory postsynaptic

potential V_S^e in relay neurons, applying the Wiener-Khinchine theorem yields the following EEG power spectrum

$$P_E(\omega) = 2\kappa\sqrt{2\pi}\left|\tilde{G}_{1,5}(\omega)\right|^2, \tag{4}$$

where

$$\tilde{\boldsymbol{G}}(\omega) = \frac{1}{\sqrt{2\pi}}\begin{bmatrix} \hat{L}_e(\omega) - K_1 & K_1 & 0 & 0 & -K_2 e^{-i\omega\tau_{CT}} & K_2 e^{-i\omega\tau_{CT}} & 0 \\ 0 & \hat{L}_i(\omega) & -f_C(p)K_3 & f_C(p)K_3 & 0 & 0 & 0 \\ -K_4 & K_4 & \hat{L}_e(\omega) & 0 & 0 & 0 & 0 \\ 0 & 0 & -K_5 & \hat{L}_i(\omega) + K_5 & 0 & 0 & 0 \\ -K_6 e^{-i\omega\tau_{TC}} & K_6 e^{-i\omega\tau_{TC}} & 0 & 0 & \hat{L}_e(\omega) & 0 & 0 \\ 0 & 0 & 0 & 0 & 0 & \hat{L}_i(\omega) & -f_T(p)K_7 \\ -K_8 e^{-i\omega\tau_{TC}} & K_8 e^{-i\omega\tau_{TC}} & 0 & 0 & -K_9 & K_9 & \hat{L}_e(\omega) \end{bmatrix}^{-1}. \tag{5}$$
