# Supplementary material for: Anesthetic action on the transmission delay between cortex and thalamus explains the beta-buzz observed under propofol anesthesia
Source: PLoS One. 2017 Jun 16;12(6):e0179286. doi: 10.1371/journal.pone.0179286 (PMC5473556; doi:10.1371/journal.pone.0179286)
Supplement: S3 Appendix — (PDF) [file pone.0179286.s005.pdf]

S3 Appendix. Finding the system characteristic roots.

In order to obtain numerically the roots of characteristic equation given by

$$det\left(\lambda\boldsymbol{I} - \boldsymbol{A} - \boldsymbol{B}e^{-\lambda\tau}\right) = 0,$$

where $\boldsymbol{A}$ and $\boldsymbol{B}$ are constant quadratic matrices of order N, and $\mathbf{I}$ is an $N \times N$ identity matrix, we have used the spectral discretization approach known as discretization of the PDE-representation of DDEs [1, 2]. In this method, the solutions of a transcendental equation coincide with the spectrum of a operator, the so-called infinitesimal generator. The discretization scheme based on a Chebyshev nodes can be implemented in three lines of MATLAB code as

```
Nod=100; N=length(A);
D=-cheb(Nod-1)*2/tau;
lambda=eig([kron(D(1:Nod-1,:),eye(N));[B,zeros(N,(Nod-2)*N), A]])
```

where "Nod" denotes the discretization nodes. The code above uses the function cheb.m, which returns a Chebyshev differentiation matrix. This function is publicly available in Ref.[3].

References

[1] A. Bellen, S. Maset, Numerical solution of constant coefficient linear delay differential equations as abstract cauchy problems, Numer. Math 84 (2000) 351–374.

[2] D. Breda, S. Maset, R. Vermiglio, Pseudospectral approximation of eigenvalues of derivative operators with non-local boundary conditions, Appl. Numer. Math. 27 (1) (2006) 318–331.

[3] L. N. Trefethen, Spectral Methods in MATLAB, Oxford Univ., Oxford, UK, 2000.
