# Supplementary material for: Anesthetic action on the transmission delay between cortex and thalamus explains the beta-buzz observed under propofol anesthesia
Source: PLoS One. 2017 Jun 16;12(6):e0179286. doi: 10.1371/journal.pone.0179286 (PMC5473556; doi:10.1371/journal.pone.0179286)
Supplement: S4 Appendix — (PDF) [file pone.0179286.s006.pdf]

S4 Appendix. Constrains on system stability.

Consider the denominator of the Fourier transform of system Green's function (cf. Eq (5) in S2 Appendix). The characteristic equation of the system can be written in the general form of transcendental equations

$$P(\lambda) + e^{-\lambda\tau} Q(\lambda) = 0, \tag{1}$$

where $P(\lambda)$ and $Q(\lambda)$ are polynomials in $\lambda = i\omega$, given by

$$P(\lambda) = a_3\lambda^3 + a_2\lambda^2 + a_1\lambda + a_0, \tag{2}$$

$$Q(\lambda) = b_2\lambda^2 + b_1\lambda + b_0, \tag{3}$$

where

$$\begin{aligned} a_3 &= \frac{1}{\beta_e^2\beta_i}, & a_2 &= \frac{2}{\beta_e\beta_i} + \frac{1}{\beta_e^2} \ , \\ a_1 &= \frac{C_2+2}{\beta_e} + \frac{1}{\beta_i}, & a_0 &= 1 + C2 \ , \\ b_2 &= 0 \ , & b_1 &= -\frac{C_1}{\beta_i} \ , \\ b_0 &= C_3 - C_1 \ . \end{aligned}$$

In the absence of delay, i.e., when $\tau = 0$, the characteristic equation described by Eq (1) recasts to

$$a_3\lambda^3 + a_2\lambda^2 + (a_1 + b_1)\lambda + (a_0 + b_0) = 0 \ . \tag{4}$$

By Routh-Hurwitz conditions, this characteristic equation is stable if and only if

$$a_3 > 0 \ , \quad a_2 > 0 \ , \quad a_1 + b_1 > 0 \ , \quad a_0 + b_0 > 0 \ , \quad a_2(a_1 + b_1) - a_3(a_0 + b_0) > 0 \ .$$

hence the non trivial conditions for the stability of the system in the absence of delay are

$$\beta_i(C_2 + 2) + \beta_e(1 - C_1) > 0, \tag{5}$$

$$C_3 + C_2 - C_1 + 1 > 0, \tag{6}$$

$$(2\beta_e + \beta_i)\left(\frac{C_2+2}{\beta_e} + \frac{1 - C_1}{\beta_i}\right) - (C_3 + C_2 - C_1 + 1) > 0. \tag{7}$$

Moreover, by squaring and adding the real and imaginary parts of the polynomials $P(\lambda)$ and $Q(\lambda)$ while the exponential is defined in terms of trigonometric functions, the characteristic polynomial $\mathcal{P}(\Omega)$ is obtained as

$$a_3^2\Omega^3 + (a_2^2 - 2a_3a_1)\Omega^2 + (a_1^2 - 2a_2a_0 - b_1^2)\Omega + (a_0^2 - b_0^2) = 0, \qquad (8)$$

where $\Omega = \omega^2$. Since $a_3 > 0$,

$$\mathcal{P}(\Omega) \equiv \Omega^3 + \xi_2\Omega^2 + \xi_1\Omega + \xi_0 = 0, \qquad (9)$$

where

$$\begin{aligned}
\xi_2 &= \frac{a_2^2 - 2a_3a_1}{a_3^2} = (2\beta_e + \beta_i)^2 - 2\left(\beta_2^2 + \beta_e\beta_i(C_2+2)\right), \\
\xi_1 &= \frac{a_1^2 - 2a_2a_0 - b_1^2}{a_3^2} = \left(\beta_e^2 + \beta_e\beta_i(C_2+2)\right)^2 - 2\beta_e^2\beta_i(2\beta_e+\beta_i)(C_2+1) - \beta_e^2C_1^2, \\
\xi_0 &= \frac{a_0^2 - b_0^2}{a_3^2} = (\beta_e^2\beta_i)^2\left((C_2+1)^2 - (C_3 - C_1)^2\right).
\end{aligned}$$

Now we seek the conditions under which the characteristic polynomial $\mathcal{P}(\Omega)$ has no positive real root. In the simplest case, if all the coefficients of a polynomial are positive, the polynomial equation can not have any positive real roots. In this situation, if the discriminant $\Delta = 18\xi_2\xi_1\xi_0 - 4\xi_2^3\xi_0 + \xi_2^2\xi_1^2 - 4\xi_1^3 - 27\xi_0^2$ is zero or positive ($\Delta \geq 0$), all the three roots are real and negative. In another situation, the above characteristic polynomial can have one root with negative real part and two complex conjugate roots. By Descartes' rule of signs, it can be shown that if the lead coefficient of a third order characteristic polynomial is positive, one negative real root is guaranteed if and only if the constant coefficient is positive. Moreover, if the discriminant $\Delta < 0$, then the polynomial equation has one real root and two complex conjugate roots. Thus, $\Delta < 0$ and $\xi_0 > 0$ guarantee that we have one negative real root and two complex roots i.e., $\mathcal{P}(\Omega)$ has not any positive real roots.

In summary, the following conditions guarantee that the system is stable in the absence of the delay, and the introduction of a time delay can not cause a bifurcation and hence, for $\tau \geq 0$, the reduced thalamo-cortical model exhibits stable oscillations:

$$
\begin{aligned}
\beta_i(C_2+2)+\beta_e(1-C_1) &> 0, \quad \text{(Condition I)}\\
C_3+C_2-C_1+1 &> 0, \quad \text{(Condition II)}\\
(2\beta_e+\beta_i)\left(\frac{C_2+2}{\beta_e}+\frac{1-C_1}{\beta_i}\right)-(C_3+C_2-C_1+1) &> 0, \quad \text{(Condition III)}\\
(\beta_e^2\beta_i)^2\left((C_2+1)^2-(C_3-C_1)^2\right) &> 0, \quad \text{(Condition IV)}\\
\Delta<0, \quad or \quad if \quad \Delta\geq 0 \; then \; \xi_1,\xi_2 &> 0. \quad \text{(Condition V)}
\end{aligned}
$$
